# A *Trichomonas vaginalis* Rhomboid Protease and Its Substrate Modulate Parasite Attachment and Cytolysis of Host Cells

**DOI:** 10.1371/journal.ppat.1005294

**Published:** 2015-12-18

**Authors:** Angelica M. Riestra, Shiv Gandhi, Michael J. Sweredoski, Annie Moradian, Sonja Hess, Sinisa Urban, Patricia J. Johnson

**Affiliations:** 1 Department of Microbiology, Immunology, and Molecular Genetics, University of California, Los Angeles, Los Angeles, California, United States of America; 2 Department of Molecular Biology and Genetics, Johns Hopkins University School of Medicine, Baltimore, Maryland, United States of America; 3 Proteome Exploration Laboratory, Division of Biology and Biological Engineering, Beckman Institute, California Institute of Technology, Pasadena, California, United States of America; 4 Howard Hughes Medical Institute, Johns Hopkins University School of Medicine, Baltimore, Maryland, United States of America; 5 Molecular Biology Institute and Department of Microbiology, Immunology, and Molecular Genetics, University of California, Los Angeles, Los Angeles, California, United States of America; University of Virginia Health System, UNITED STATES

## Abstract

*Trichomonas vaginalis* is an extracellular eukaryotic parasite that causes the most common, non-viral sexually transmitted infection worldwide. Although disease burden is high, molecular mechanisms underlying *T*. *vaginalis* pathogenesis are poorly understood. Here, we identify a family of putative *T*. *vaginalis* rhomboid proteases and demonstrate catalytic activity for two, TvROM1 and TvROM3, using a heterologous cell cleavage assay. The two *T*. *vaginalis* intramembrane serine proteases display different subcellular localization and substrate specificities. TvROM1 is a cell surface membrane protein and cleaves atypical model rhomboid protease substrates, whereas TvROM3 appears to localize to the Golgi apparatus and recognizes a typical model substrate. To identify TvROM substrates, we interrogated the *T*. *vaginalis* surface proteome using both quantitative proteomic and bioinformatic approaches. Of the nine candidates identified, TVAG_166850 and TVAG_280090 were shown to be cleaved by TvROM1. Comparison of amino acid residues surrounding the predicted cleavage sites of TvROM1 substrates revealed a preference for small amino acids in the predicted transmembrane domain. Over-expression of TvROM1 increased attachment to and cytolysis of host ectocervical cells. Similarly, mutations that block the cleavage of a TvROM1 substrate lead to its accumulation on the cell surface and increased parasite adherence to host cells. Together, these data indicate a role for TvROM1 and its substrate(s) in modulating attachment to and lysis of host cells, which are key processes in *T*. *vaginalis* pathogenesis.

## Author Summary

*Trichomonas vaginalis*, a common pathogen with a worldwide distribution, causes a sexually transmitted infection and exacerbates other diseases. Estimated to infect over a million people annually in the United States alone, the Center for Disease Control and Prevention categorized trichomoniasis as one of five neglected parasitic diseases in the US in 2014. Only one class of drug is available to treat *T*. *vaginalis* infection, making discovery of parasite factors contributing to host colonization critical for the development of new therapeutics. Here we report the first characterization of *T*. *vaginalis* intramembrane rhomboid proteases. One protease, TvROM1, is shown to increase the parasite’s association with and destruction of host cells. We further identified two TvROM1 substrates, one of which we demonstrate is involved in modulating host: parasite interactions. This study highlights the involvement of rhomboid proteases in *T*. *vaginalis* pathogenic processes, and provides further support for targeting parasite surface proteases for therapeutic intervention.

## Introduction

*Trichomonas vaginalis* is an extracellular, eukaryotic parasite that is the causative agent of trichomoniasis, the most common non-viral sexually transmitted infection in the world [1]. Approximately 276 million people worldwide become newly infected each year [1]. In the United States, an estimated 3.7 million people are currently infected [2]. Symptoms and outcomes of infection include vaginitis, urethritis, prostatitis, infertility, and adverse pregnancy outcomes (reviewed in Petrin *et al*. 1998) [3, 4]. *T*.*vaginalis* infection is associated with an increased risk of HIV acquisition [5] and potential transmission [6] due to HIV target cell recruitment to the site of infection [7] and increased viral shedding upon co-infection [8, 9]. *T*. *vaginalis* is also associated with cervical cancer [10, 11] and aggressive prostate cancer [12, 13]. Due to the high burden, threat of illness, and understudied nature of *T*. *vaginalis* infection, trichomoniasis has been recently recognized as one of the United States’ neglected parasitic infections [4, 14, 15].

Although the magnitude of parasite infection is high, little is known about how *T*. *vaginalis* colonizes the human host and causes disease [3, 16]. As an extracellular organism that thrives in the changing and physiologically diverse environment of the urogenital tract of men and women, *T*. *vaginalis* likely utilizes multiple mechanisms to establish an infection and persist. The parasite attaches to multiple host cell types such as vaginal and prostate epithelial cells [17], red blood cells [18], and is capable of acquiring nutrients from them through host cell lysis. *T*. *vaginalis* can also bind together to form clusters [19]. Parasite cell aggregates are observed upon axenic growth and when placed on monolayers of host cells. However, only a few of the molecular players that mediate and regulate these parasite-parasite or host-parasite interactions have been identified [16, 19, 20].

Recent genomic, transcriptomic, and proteomic studies of *T*. *vaginalis* have aided the identification of protein families that may play important roles in *T*. *vaginalis* cell biology and pathogenesis [21–24]. In studies analyzing the surface proteome of *T*. *vaginalis*, we identified a rhomboid-like protein [23]. Rhomboid proteases are polytopic serine proteases that are localized to membranes where they encounter and cleave their substrates. Thus they belong to the intramembrane-cleaving protease (I-CLiPs) class of enzymes [25]; however, rhomboid proteases differ from most I-CLiPs in that their cleavage products are often released to the outside of the cell. Rhomboid proteases were first described in *Drosophila melanogaster*, where they shed growth factors from the membrane to initiate cell signaling in neighboring cells [26–28].

Rhomboid proteases are among the most conserved families of polytopic membrane proteins [29] and have been suggested to be one of the most ancient regulatory enzymes [25]. In addition to activating cell signaling pathways, rhomboid proteases have been reported to play roles in quorum sensing [30, 31], regulation of mitochondrial morphology [32], and phagocytosis [33]. Moreover, in intracellular parasites like *Toxoplasma* [34–38] and *Plasmodium* [39–43] as well as the extracellular parasite *Entamoeba histolytica* [44] rhomboid proteases cleave parasite adhesins.

One of the greatest challenges in uncovering the biological functions of rhomboid proteases has been the identification of their substrates. For example, the substrate for the most studied bacterial rhomboid, *Escherichia coli* GlpG, is still unknown [45]. Rhomboid proteases from evolutionarily divergent organisms are capable of recognizing and cleaving a common set of substrates [26, 31, 34, 39, 44, 46]. This property has allowed the identification of active rhomboid proteases, from diverse organisms, in the absence of a known substrate.

Rhomboid proteases and other I-CLiPs evolved separately from soluble proteases [25], hence the mechanism(s) they use for substrate recognition and cleavage is also predicted to be different, as recent studies have begun to reveal [47–49]. Selection of a specific rhomboid substrate is dictated by transmembrane (TM) segment dynamics of the substrate [49]. In particular, helix-destabilizing residues in the TM domain appear to be required for the substrate to exit the membrane and reside in the enzyme’s inner active site [49]. There is also a preference for certain amino acids surrounding the cleavage site. Cleavage of the substrate occurs between small amino acids at positions designated P1 and P1’ [46, 50, 51]. Additional preferences for particular amino acids at the P4 and P2’ sites for the eubacterial AarA rhomboid led to the prediction of a universal recognition motif for substrate binding by rhomboid proteases [46]. However, further studies revealed that most eukaryotic rhomboid proteases do not conform to this prediction [49]. The discovery of additional substrates will aid in defining specific substrate features that promote rhomboid protease cleavage and how these may differ between different classes of rhomboid proteases.

In this study, we report the identification and characterization of two active *T*. *vaginalis* rhomboid proteases (TvROM1 & TvROM3). Exogenous expression of TvROM1 increases attachment to and cytolysis of host ectocervical cells, which are two phenotypes implicated in pathogenesis. Using quantitative proteomics and bioinformatics, we identified and verified two TvROM1 substrates, TVAG_166850 and TVAG_280090. Neither TvROM1 substrate is cleaved by TvROM3, hence the two enzymes have different natural substrates, consistent with their specificity for different model substrates and different subcellular localizations. Importantly, the *T*. *vaginalis* substrates identified for TvROM1 belong to a family of putative adhesins previously implicated in pathogenesis [20, 23]. Together, our observations demonstrate a role for TvROM1 and its substrate(s) in modulating parasite attachment and cytolysis of host cells.

## Results

### Identification of active rhomboid proteases in *T*. *vaginalis*

To assess the function of rhomboid proteases in the extracellular parasite *T*. *vaginalis*, we interrogated its genome (http://trichdb.org/trichdb) and found nine genes annotated as “rhomboid-like”. Bioinformatic analysis of these genes revealed that only four encode both a serine (Ser) and a histidine (His) at the top of TMs 4 and 6 which are predicted to form the catalytic dyad for substrate cleavage (S1 Fig). The proteins encoded by these four genes also have the Gly-X-Ser-X motif, (X = amino acid) that surrounds and identifies the catalytic serine residue [52]. We named these putative active *T*. *vaginalis* rhomboid proteases TvROM1-4 (TrichDB accession numbers TVAG_112900, TVAG_359500, TVAG_476950, TVAG_161010). The other five gene products (TrichDB accession numbers TVAG_282180, TVAG_378960, TVAG_233140, TVAG_183030, TVAG_037580) lack one or both catalytic residues and are likely proteolytically inactive. TVAG_233140, which lacks a catalytic His, was detected in the surface proteome of *T*. *vaginalis* [23]. We focused our attention on the rhomboid proteins predicted to be active proteases. S1 Fig summarizes additional features found in the four predicted active *T*. *vaginalis* rhomboid proteases. The alignment in S1 Fig also reveals differences in the N- and C-termini amongst TvROMs 1–4, a feature that has been previously noted when comparing rhomboid protease sequences from multiple organisms [52].

### Localization of TvROMs in *T*. *vaginalis*

**Fig 1:**
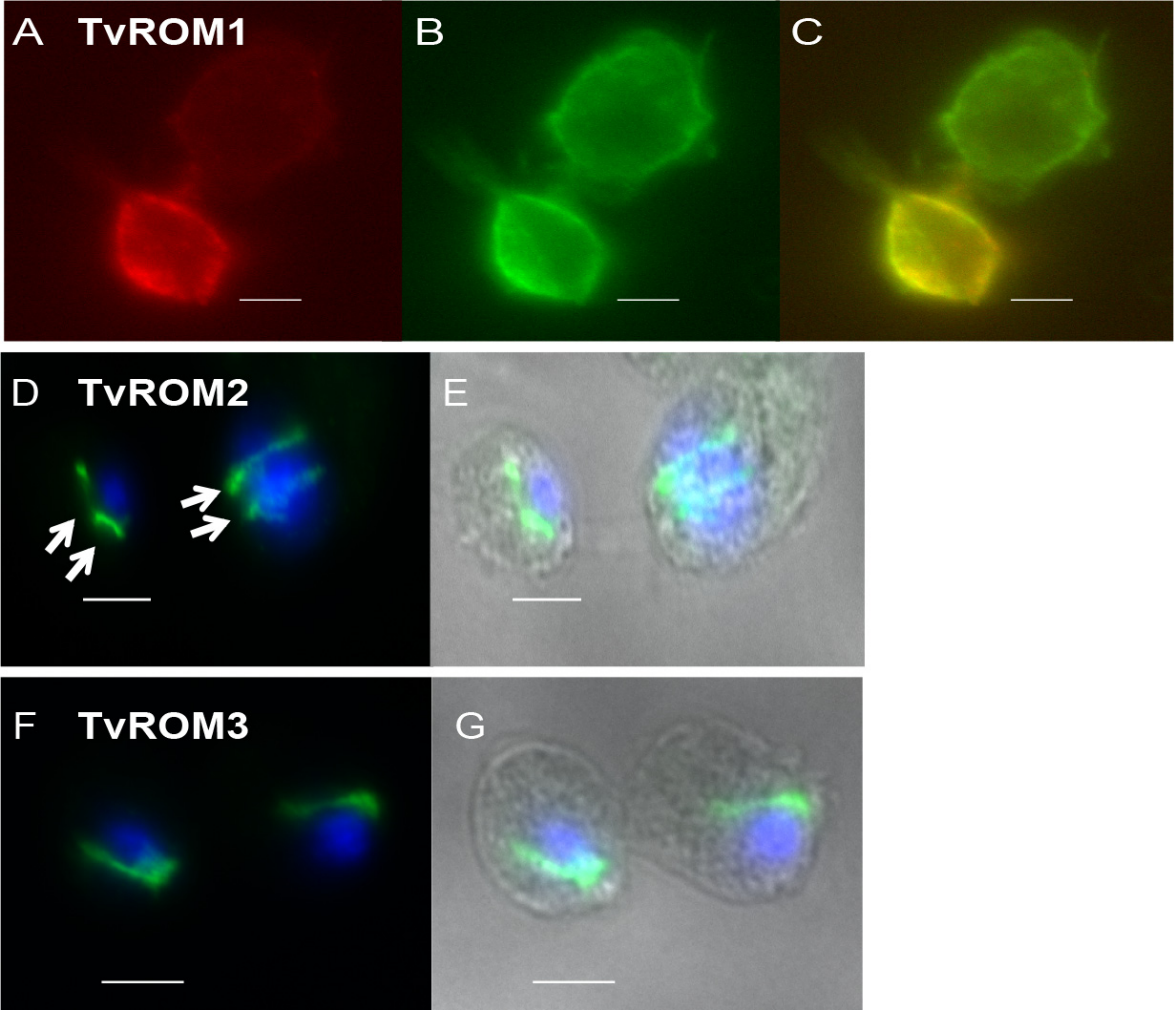
Subcellular localization of HA-tagged TvROMs in *T*. *vaginalis* transfectants. Fluorescence microscopy images of indirect immunofluorescence assays (IFA) performed on *T*. *vaginalis* exogenously expressing N-terminal hemagglutinin (HA)-tagged TvROMs 1–3. (**A-C**) HA-TvROM1 transfectants were reacted with membrane impermeable biotin (EZ-Link-Sulfo-NHS-SS-Biotin) and IFA was then performed using formaldehyde fixation and staining with rabbit anti-HA (A-red) and a mouse anti-Biotin (B-green) antibodies. (**C**) Merge shows co-localization of HA-TvROM1 with the biotin-labeled *T*. *vaginalis* cell surface, scale bar = 10 μm. IFA images of HA-TvROM2 (**D & E**) and HA-TvROM3 (**F & G**) using a mouse anti-HA antibody (green) and nuclear staining using 4’-6’-diamidino-2-phenylindole (DAPI-blue). HA-TvROM2 and HA-TvROM3 show localization in a line structure adjacent to the nucleus, scale bar = 5 μm. Two juxtanuclear structures of different sizes can be observed in early and late dividing cells in HA-TvROM2 transfectants (**D**, see arrows). Phase images are shown on the right.

The genes encoding the predicted active rhomboids were cloned under the control of the strong α-SCS promoter with N-terminal hemagglutinin (HA) tags and transfected into *T*. *vaginalis*. TvROMs were tagged at their N-terminus based on reports that HA-N-terminally-tagged rhomboid proteases localize properly and are active, while addition of a C-terminal tag can abolish proteolytic activity [34, 39, 44]. Furthermore, all TvROMs lack predicted N-terminal signal peptides as analyzed by several signal peptide and membrane topology prediction programs. As has been observed for other multi-spanning membrane proteins, TvROMs 1–4 are predicted to be properly targeted and inserted in the membrane via their first TM domain [53]. The subcellular localization of the proteins was determined in transfectants using indirect immunofluorescence assays (IFA) with an anti-HA antibody. Initial analyses indicated a cell surface localization of HA-TvROM1. To confirm this localization, transfectants were labeled using membrane-impermeable biotin as described previously [23] and a co-localization of the HA-TvROM1 signal with the biotinylated cell surface was observed (Fig 1A–1C). No background staining was observed in non-biotinylated parasites used as a negative control.

In contrast to the cell surface localization of HA-TvROM1, HA-TvROM2 and HA-TvROM3 were located in a line structure next to the nucleus (Fig 1D, 1E, 1F, and 1G, respectively), which is likely the Golgi apparatus. Many rhomboid proteases have been localized in the Golgi apparatus, including the founding member of the rhomboid family, *Drosophila* Rhomboid-1 (DmRho1) [26] and the *Toxoplasma gondii* TgROM2 [54]. Prior to mitosis, the *T*. *vaginalis* Golgi apparatus elongates and then divides through medial fission of each cisternae in a process called Golgikinesis, forming two Golgi ribbons [55]. We observed a similar pattern of staining adjacent to the nucleus in dividing cells where HA-TvROM2 (Fig 1D and 1E) and HA-TvROM3 localize, providing additional support for a Golgi localization of these proteins. We were unable to detect expression of HA-TvROM4 in transfectants and therefore did not continue to analyze this protein.

### TvROM1 and TvROM3 are active proteases

We tested the proteolytic activities of TvROM1-3 using an established heterologous cell cleavage assay [39]. Synthetic open reading frames of each TvROM were designed to match codon bias of human cells to facilitate expression in human cells. These proteases were HA-tagged and co-expressed in HEK293 cells with model rhomboid protease substrates tagged at the N-terminus with GFP or doubly tagged with an N-terminal GFP tag and a C-terminal FLAG tag. Cleavage was then assessed by detection of released GFP-tagged substrates of the predicted size. Rhomboid proteases known to cleave the model substrate being tested were also co-transfected with the substrate as a positive control. Cells transfected only with the model substrate or co-transfected with catalytically inactive rhomboid mutants served as negative controls.

**Fig 2:**
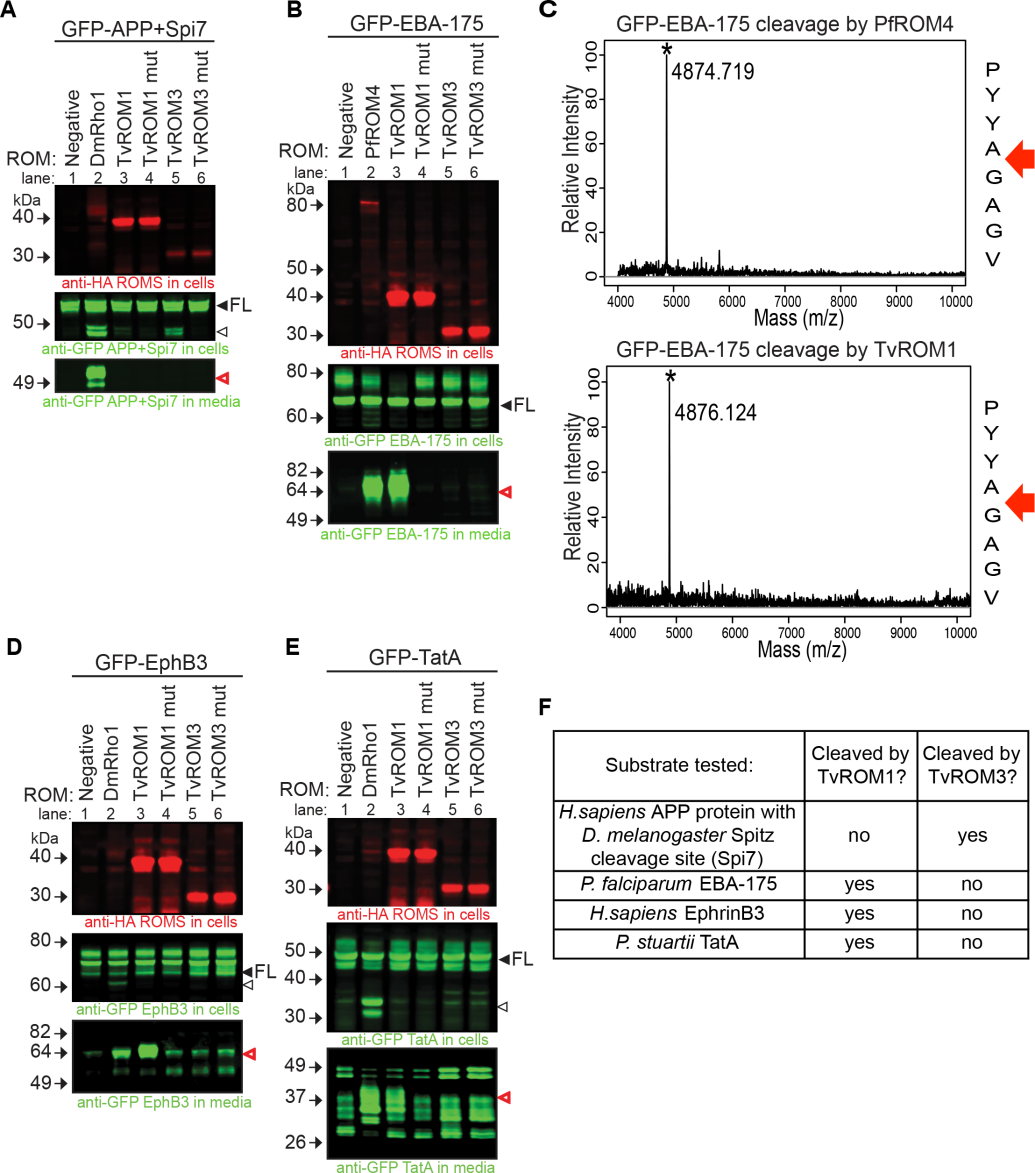
TvROM catalytic activity analyses. The activity of *T*. *vaginalis* rhomboid proteases were tested by co-transfecting the proteases with known model rhomboid substrates in a heterologous cell cleavage assay using HEK293 cells. Proteases were HA tagged and substrates contained an N-terminal GFP tag to allow detection. **(A, B, D and E)** Whole cell lysates (WCL) and conditioned media (CM) collected from co-transfectants was analyzed by Western blot analyses [39]. **Top panels**: rhomboid protease detected in WCL using an anti-HA antibody; **middle panels**: full-length (FL, filled arrowheads) and cleaved substrates (open arrowheads) detected in WCL using an anti-GFP antibody; **bottom panels**: cleaved substrate fragments detected in CM using an anti-GFP antibody (open, red arrowheads). The substrates tested were **(A)** APP+7 residues of the *Drosophila melanogaster* Spitz protein encompassing the rhomboid protease cleavage site (APP+Spi7), **(B)** *Plasmodium falciparum* EBA-175, **(D)** human EphB3, and **(E)** *Providencia stuartii* TatA. The positive control HA-tagged rhomboid protease (lane 2) used for cleavage of Spitz **(A)**, human EphB3 **(D)**, and TatA **(E)** was DmRho1; the positive control protease for cleavage of EBA-175 **(B)** was HA-tagged PfROM4 (lane 2). Negative controls (lane 1) were transfected with only substrates. TvROM1/TvROM3 = wild type protease; TvROM1 mut/TvROM3 mut = protease with the catalytic histidine mutated to alanine. TvROM3 was found to cleave only the Spitz TM domain segment of APP+Spi7 (**A** middle panel, lane 5) whereas TvROM1 does not cleave the Spitz sequence (**A** bottom panel, lane 3) but does cleave the other 3 substrates (**B, D,** and **E** bottom panels, lane 3). **(F)** Summary of the cleavage data shown in panels A, B, D, and E. **(C)** The location of EBA-175 cleavage by PfROM4 (control; top panel) and TvROM1 (bottom panel) determined by subjecting the immunoprecipitated cleavage fragment to MALDI-TOF analysis. Red arrows indicate that both proteases cleave the substrate between the two small amino acids alanine (A) and glycine (G).

We first tested whether TvROMs cleave the *D*. *melanogaster* rhomboid substrate Spitz [26], a canonical rhomboid substrate cleaved by rhomboid proteases from most organisms tested [34, 39, 44, 51, 56]. We discovered that TvROM3 was the only TvROM that could consistently cleave Spitz, albeit weakly. TvROM3 cleavage was more efficient with APP+Spi7, a chimeric substrate in which the first 7 residues of the amyloid precursor protein (APP) TM segment were replaced with those from Spitz (Fig 2A-lane 5). The positive control DmRho1 cleaved APP+Spi7 (Fig 2A-lane 2) releasing the cleaved fragment into the media. Cleavage by TvROM3 was specific as no cleavage product was detected with the TvROM3 His181Ala mutant (Fig 2A-lane 6).

*Plasmodium falciparum* rhomboid 4 (PfROM4) displays ‘atypical’ specificity, because it cannot process Spitz yet cleaves many *Plasmodium* adhesins [39]. Since TvROM1 was unable to cleave Spitz, we tested its ability to cleave *Plasmodium* adhesins erythrocyte-binding antigen 175 (EBA-175) protein (Fig 2B) and the BA erythrocyte binding ligand (BAEBL/EBA140) protein (S2A Fig). TvROM1 cleaved EBA-175 (Fig 2B-lane 3) and BAEBL (S2A Fig -lane 3) efficiently. Conversely, the TvROM1 His316Ala mutant did not cleave these two proteins (Fig 2B-lane 4 and S2A Fig -lane 4). The ability of TvROM1 to cleave *Plasmodium* adhesins but not Spitz protein categorizes it as having atypical substrate specificity [39]. Importantly, in addition to PfROM4 and *Entamoeba histolytica* rhomboid 1 (EhROM1), TvROM1 is the third parasite rhomboid protease that displays atypical substrate specificity [39, 44] suggesting this may be a common feature of eukaryotic parasite rhomboid proteases. Since TvROM1 displayed similar substrate specificity as PfROM4, we determined whether TvROM1 also cleaves EBA-175 at the same site as PfROM4. The FLAG-tagged C-terminal cleavage product produced by TvROM1 and PfROM4 co-transfectants was immunoprecipitated and the cleavage site was determined by MALDI-TOF analysis. We found that TvROM1 cleaves EBA-175 between Ala and Gly (Fig 2C) as previously found for PfROM4 [42]. Therefore, TvROM1 also cleaves its substrate between two small amino acids.

TvROM1 could also cleave human EphrinB3 (Fig 2D-lane 3), and bacterial TatA proteins (Fig 2E-lane 3), which are substrates of the human RHBDL2 [57] and the bacterial AarA [30] rhomboid proteases, respectively. DmRho1 served as a positive control in the EphrinB3 and TatA cleavage assays (Fig 2D-lane 2 and Fig 2E-lane 2, respectively). Interestingly, although DmRho1 cleaves Spitz and these additional substrates, TvROM1 cleaved EphrinB3 and TatA, but not Spitz. Cleavage of EphrinB3 and TatA by TvROM1 was specific, since the TvROM1 His316Ala mutant did not produce cleavage fragments (Fig 2D-lane 4 and Fig 2E-lane 4). Conversely, TvROM3 did not specifically cleave EBA-175 (Fig 2B- lanes 5 and 6), EphrinB3 (Fig 2D-lanes 5 and 6), or TatA (Fig 2E-lanes 5 and 6). Taken together, these results identify different substrate specificities for TvROM1 and TvROM3 (summarized in Fig 2F).

TvROM2 produced cleavage fragments of Spitz (S2D Fig-lane 4), however, the fragments were also produced by the TvROM2 His270Ala mutant (S2D Fig -lane 5). As endogenous metalloproteases have been shown to cleave and release rhomboid substrates in the heterologous cell cleavage assay [26, 34, 39], we added a wide-spectrum metalloprotease inhibitor (10 μM batimastat-BB-94) in assays testing whether TvROM2 cleaves EphrinB3 (S2B Fig). The inhibitor greatly reduced the appearance of the predicted EphrinB3 cleavage fragment produced by TvROM2 (S2B Fig-lane 3 *vs*. lane 7) and the TvROM2 mutant (S2B Fig-lane 4 *vs*. lane 8) but not by TvROM1 (S2B Fig -lane 2 *vs*. 6) that we used as a positive control. Therefore the cleavage products observed with the other substrates in the TvROM2 co-transfectants (S2D and S2E Fig, lanes 4 and 5) are also likely due to endogenous metalloprotease activity and not rhomboid protease activity.

### Serine protease activity and TvROM1 contributes to *T*. *vaginalis* attachment to and cytolysis of host ectocervical cells

**Fig 3:**
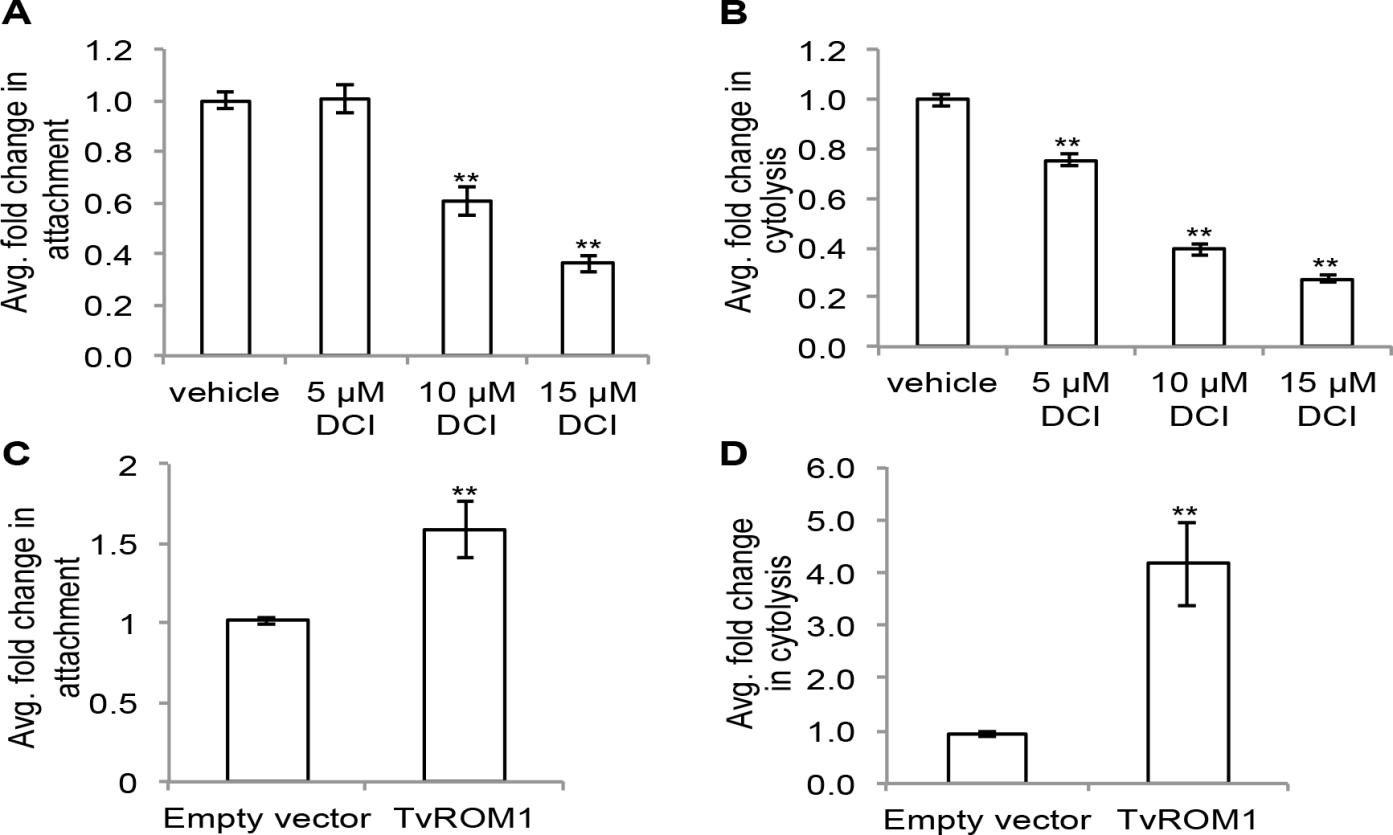
Serine protease activity and TvROM1 contribute to *T*. *vaginalis* attachment and lysis of ectocervical cells. **(A)** Fluorescently labeled *T*. *vaginalis* incubated with ectocervical cell monolayers in the presence of increasing concentrations of the serine protease inhibitor 3,4-dichloroisocoumarin (3,4-DCI) followed by quantification of adhered parasites. The average fold change in attachment compared to vehicle control for four experiments each performed in triplicate is shown. Error bars denote the standard error, **p<0.01. **(B)** Parasites incubated with ectocervical cell monolayers in the presence of increasing 3,4-DCI followed by assessment of ectocervical cell lysis. The average fold change in cytolysis compared to vehicle control for three experiments performed in triplicate is shown. Error bars denote the standard error, **p<0.01. **(C)** Average fold difference in attachment of HA-TvROM1 transfectants compared to empty vector transfectants shown for four experiments each conducted in triplicate, with standard error shown as error bars, **p<0.01. **(D)** Average fold change in cytolysis of ectocervical cells by HA-TvROM1 transfectants compared to empty vector transfectants, results are from four experiments performed in triplicate. Standard error is shown as error bars, **p<0.01.

To investigate the biological role of TvROMs and their possible contribution to parasite-host interactions, we tested the effect of the serine protease inhibitor 3,4-dichloroisocoumarin (3,4-DCI) on parasite attachment to and lysis of host cells. 3,4-DCI has been shown to inhibit the activity of rhomboid proteases from multiple organisms [33, 34, 40, 58]. The effect of 3,4-DCI on parasite attachment to host cells was tested by exposing *T*. *vaginalis* to ectocervical cell monolayers for 30 min in the presence of increasing concentrations of 3,4-DCI. Unattached parasites were then washed away and the number of attached parasites was quantified (as previously described [17]). A dose-response effect was observed even with low concentrations of 10 and 15 μM 3,4-DCI, which decreased attachment to host cells 40% and 64%, respectively, compared to vehicle-treated parasites (Fig 3A). To test the effect of 3,4-DCI on cytolysis of host cells, we exposed *T*. *vaginalis* to ectocervical cell monolayers for three hours in the presence of different 3,4-DCI concentrations and then assessed host cell lysis by measuring LDH released from damaged cells as described [17]. Similar to the observed reduction in attachment, cytolysis of host cells was reduced by 25%, 61% and 73% in the presence of 5, 10 and 15 μM 3,4-DCI, respectively (Fig 3B). No effect on the viability of the parasites or host cells was observed at the concentrations used for both the attachment and cytolysis assays. These results suggest that serine proteases likely play a role in two critical processes that contribute to *T*. *vaginalis* destruction of host cells.

To test the role of individual TvROMs specifically, we compared the ability of parasites exogenously expressing HA-tagged TvROMs to attach to and lyse host cells. Parasites expressing additional exogenous HA-TvROM1 had a 1.6-fold increase in attachment to host cells compared to empty vector control transfectants (p-value<0.01) (Fig 3C). Additionally, exogenous expression of HA-TvROM1 resulted in a 4.2-fold increase in host cell lysis (p-value<0.01) (Fig 3D). The involvement of TvROMs in these processes was limited to TvROM1, as exogenous expression of TvROM2 and TvROM3 did not lead to increased attachment to or lysis of host cells.

### TvROM1 substrate identification using quantitative proteomics

**Fig 4:**
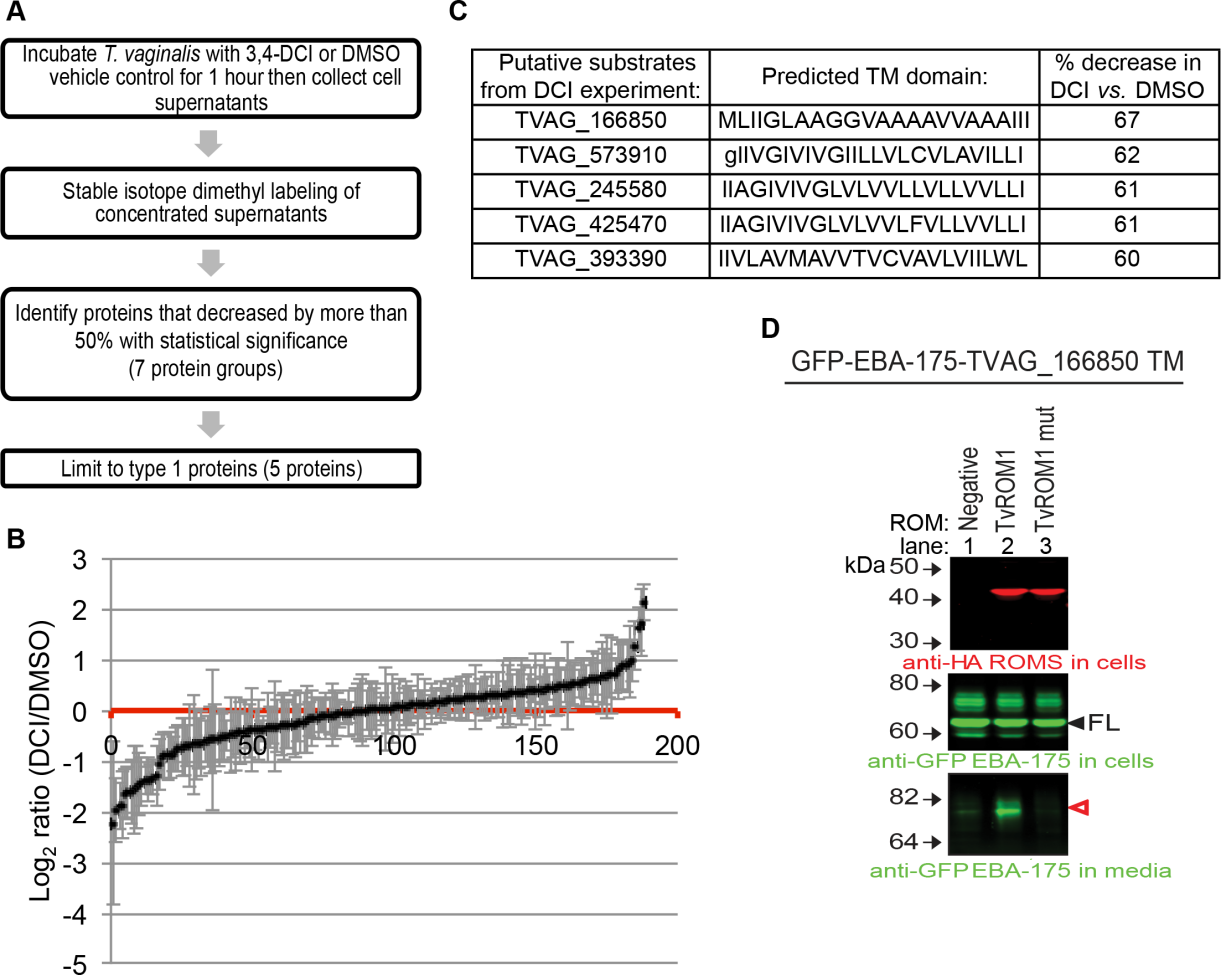
Use of quantitative proteomics identifies a putative TvROM1 substrate. **(A)** Flow chart of the approach taken to identify putative substrates for TvROM1 using quantitative proteomics of cell supernatants from HA-TvROM1 *T*. *vaginalis* transfectants treated with vehicle or 50 μM 3,4-DCI serine protease inhibitor. **(B)** The profile of proteins identified in two independent mass spectrometry experiments and the magnitude of change on a log_2_ scale, errors bars denote the standard error. Proteins that decreased with 3,4-DCI treatment have log_2_(DCI/DMSO) ratios<0, and those that increased are >0. **(C)** The predicted TM domains of the five putative substrates identified in **A**, and the percent decrease in protein levels with 3,4-DCI *vs*. DMSO vehicle treatment is shown. Capital letters indicate amino acids of the predicted TM domain, lowercase letters denote amino acids found outside the predicted TM domain. **(D)** The TM domain of TVAG_166850 is cleaved by TvROM1. A fusion protein composed of GFP-*P*. *falciparum* EBA-175 and the TVAG_166850 TM domain was co-expressed with HA-TvROM1 (lane 2) or a TvROM1 catalytic His to Ala mutant (lane 3) using the HEK293 heterologous cell cleavage assay. Negative control lacked co-transfection with a TvROM (lane 1). Western blot analysis of whole cell lysates (top and middle panels) and media (bottom panel) was performed as described in text and Fig 2 legend. The expression of TvROM1 proteins and the substrate (filled arrowhead) is confirmed in top and middle panels, respectively. Bottom panel shows release of the cleaved substrate (open, red arrowhead) specifically in TvROM1 co-transfectants. The remaining putative substrates were not cleaved by TvROM1 or TvROM3 (see S3 Fig).

Having demonstrated that TvROM1 is an active protease that modulates attachment to and lysis of host cells, we sought to identify its substrates using a quantitative proteomics approach. Given the cell surface localization of TvROM1, we identified proteins that were differentially released into the media by TvROM1 transfectants treated with the serine protease inhibitor 3,4-DCI compared to DMSO vehicle control (see flow chart in Fig 4A). Proteins released into the media were collected and differentially labeled using stable isotope dimethyl labeling [59] to allow quantitative comparisons of protein abundance, with the prediction that inhibition of TvROM1 activity would reduce the release of its plasma membrane substrates. S1 Table lists and Fig 4B shows the distribution of all the proteins identified in a combined analysis of two independent mass spectrometry experiments. Of the proteins identified, seventeen protein groups decreased by more than 50% with 3,4-DCI *vs*. vehicle control treatment, with a statistically significant decrease observed for seven protein groups (see S1 Table). Five proteins of the seven protein groups (TVAG_166850, TVAG_573910, TVAG_245580, TVAG_425470, TVAG_393390) have a predicted TM domain and type 1 topology (N-terminus outside cell-TM domain-C-terminus inside cell), which constitute the minimal features of rhomboid protease substrates. The abundance of the five candidate substrates in the *T*. *vaginalis* media was reduced 60–67% in the presence of 50 μM 3,4-DCI (Fig 4C). Mass spectrometry data from these experiments have been deposited to Chorus and are available for download (https://chorusproject.org, project ID 969).

The ability of TvROM1 to cleave the TM domains of the five candidate substrates was tested using the HEK293 heterologous cell cleavage assay. Initial attempts to express the full-length *T*. *vaginalis* substrates resulted in retention of these large proteins in the ER. Therefore, to assess cleavage at the cell surface, a chimeric protein of GFP-EBA-175 with its TM domain replaced with each of the five putative substrate’s TM domain was constructed and co-expressed with either TvROM1 or a TvROM1 His316Ala mutant. We found that only the TM domain of TVAG_166850 could be specifically cleaved by TvROM1 (Fig 4D, bottom panel, lane 2, and S3A–S3D Fig) but not the TvROM1 His316Ala mutant (Fig 4D, bottom panel, lane 3). Although we observed release of TVAG_573910 into the media, cleavage was not dependent on co-transfection with TvROM1 (S3A Fig -lanes 1 and 2). We also tested whether any of the putative substrates could be cleaved by TvROM3, the other active rhomboid protease identified (Fig 2), but TvROM3 failed to cleave any of the five putative substrates (see S3A–S3D Fig). Inspection of the amino acids in the TM domain of the five putative substrates (Fig 4C) revealed that the four proteins that were not cleaved (TVAG_573910, TVAG_245580, TVAG_425470, and TVAG_393390) contain residues at the top of the TM domain that fit the previously published Strisovsky *et al*. motif [46]. Therefore, the substrate specificity of both TvROM1 and TvROM3 appear to differ from the bacterial-like rhomboid specificity defined by the Strisovsky *et al*. motif. Instead, the TM domain of the cleaved substrate TVAG_166850, has a predominant presence of the small amino acid alanine, and the helix relaxing residue glycine [60, 61] (Fig 4C). This finding led us to investigate possible substrate determinants that might aid in identification of additional *T*. *vaginalis* rhomboid substrates.

### Screening the *T*. *vaginalis* surface proteome for TM domains similar to TMs in parasite rhomboid protease substrates identifies an additional TvROM1 substrate and reveals features of *T*. *vaginalis* substrate specificity

**Fig 5:**
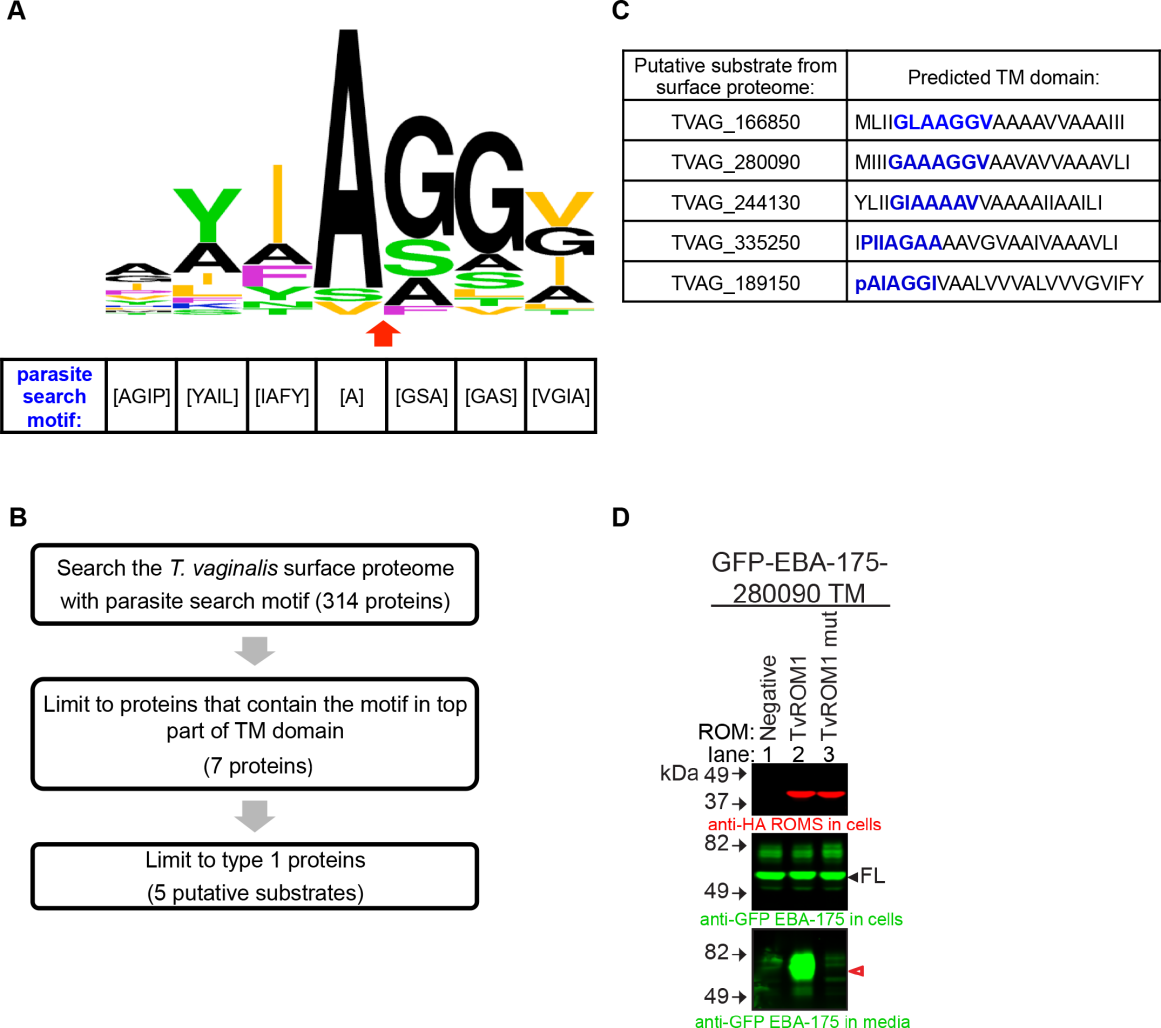
Screening of the *T*. *vaginalis* surface proteome with a parasite search motif identifies an additional TvROM1 substrate. **(A)** Graphical representation of the amino acids found in the predicted cleavage site of 20 parasite rhomboid protease substrates and the canonical *Drosophila* rhomboid substrate Spitz. The height of an amino acid indicates its relative frequency. Residue colors demark the properties of their side chains: small = black (A and G), basic = blue (K), aliphatic = orange (L, I, and V), green = uncharged, polar (Y, T, S, and N), nonpolar, nonaliphatic = purple (F and P). The predicted cleavage site is marked by a red arrow. The most common amino acids in these proteins were used to generate a “parasite search motif” (shown below). **(B)** Flow chart of the approach taken to identify putative TvROM1 substrates by searching the *T*. *vaginalis* surface proteome published by de Miguel *et al*. 2010 with the parasite search motif. **(C)** The accession numbers and predicted TM domain of the five putative surface proteome substrates are shown. The parasite search motif is indicated by blue font. Capital letters indicate amino acids of the predicted TM domain, lower case letters denote amino acids found outside the predicted TM domain. **(D)** The TM domain of putative substrate TVAG_280090 is cleaved by TvROM1. A GFP-EBA-175 chimeric protein that has its TM domain replaced with that of TVAG_280090 was co-expressed with TvROM1 or TvROM1 catalytic His to Ala mutant (mut) in the HEK293 heterologous cell cleavage assay. Negative control lacked co-transfection with a TvROM (Negative-lane 1). Western blot analyses of whole cell lysates confirm expression of the TvROM1 wt and mut proteins (top panel) and the full length substrate (filled arrowhead, middle panel). Analyses of conditioned media from the co-transfectants show a GFP-tagged cleavage product in the media (red arrowhead, bottom panel) of TvROM1 co-transfectants (TvROM1) and not in the TvROM1 catalytic His to Ala mutant (TvROM1 mut). The remaining putative substrates were not cleaved by TvROM1 or TvROM3 (see S4 Fig).

Rhomboid proteases cleave near or at the external face of the TM domain of the substrate. Since the substrate’s overall TM dynamics has been found to be the governing feature defining a rhomboid substrate [49], a subset of amino acids surrounding the cleavage site are likely to play a major role in promoting a substrate conformation that leads to cleavage [51]. Thus, we compiled and compared established and predicted cleavage sites for 20 rhomboid substrates from other eukaryotic parasites: *Toxoplasma gondii*, *Plasmodium falciparum*, and *Entamoeba histolytica* [34–37, 39, 41, 42, 44, 51, 54]. The resulting comparison revealed a preference for specific residues (Fig 5A) from which we created a consensus called the parasite search motif (Fig 5A). It is notable that this motif has little overlap with the consensus motif of the P4-P2’ sites preferred by bacterial rhomboid proteases [46].

Using the parasite search motif, we screened the *T*. *vaginalis* surface proteome [23] to identify putative TvROM1 substrates (see flowchart in Fig 5B). Of 314 proteins searched, seven proteins had the motif positioned in the top part of the TM domain by at least two of three TM prediction programs (S2 Table) and five of these were type 1, single TM proteins, typical of rhomboid protease substrates. The predicted TM domains of the five putative substrates are shown in Fig 5C. TVAG_166850, the only TvROM1 substrate identified in the quantitative proteomics approach (Fig 4D-lane 2) is also one of the five candidate substrates identified by this bioinformatics approach.

After confirming that the five candidate substrates are localized to the plasma membrane, we performed heterologous cell cleavage assays to test whether their TM domains are cleaved by TvROM1. In addition to the TM domain of TVAG_166850 (Fig 4D), we found that the TM domain of TVAG_280090 is cleaved by TvROM1 (Fig 5D-lane 2, bottom panel). It is notable that the TVAG_280090 protein has overall 55% identity and 69% similarity to TVAG_166850. The TvROM1 His316Ala mutant could not cleave TVAG_280090’s TM domain (Fig 5D-lane 3, bottom panel), demonstrating specific cleavage. DmRho1 can also process GFP-TVAG_280090 (S4F Fig -lane 4), providing further support that this protein is a rhomboid protease substrate. Similar to that observed for TVAG_166850 (S4A Fig -lane 6) TvROM3 could not cleave the TM of TVAG_280090 (S4B Fig -lane 6). None of the remaining candidate substrates were cleaved by TvROM1 or TvROM3 (S4A–S4E Fig).

### Mutation of predicted rhomboid cleavage site in the putative substrate TVAG_166850 increases its presence at the cell surface and leads to greater parasite attachment to host cells

**Fig 6:**
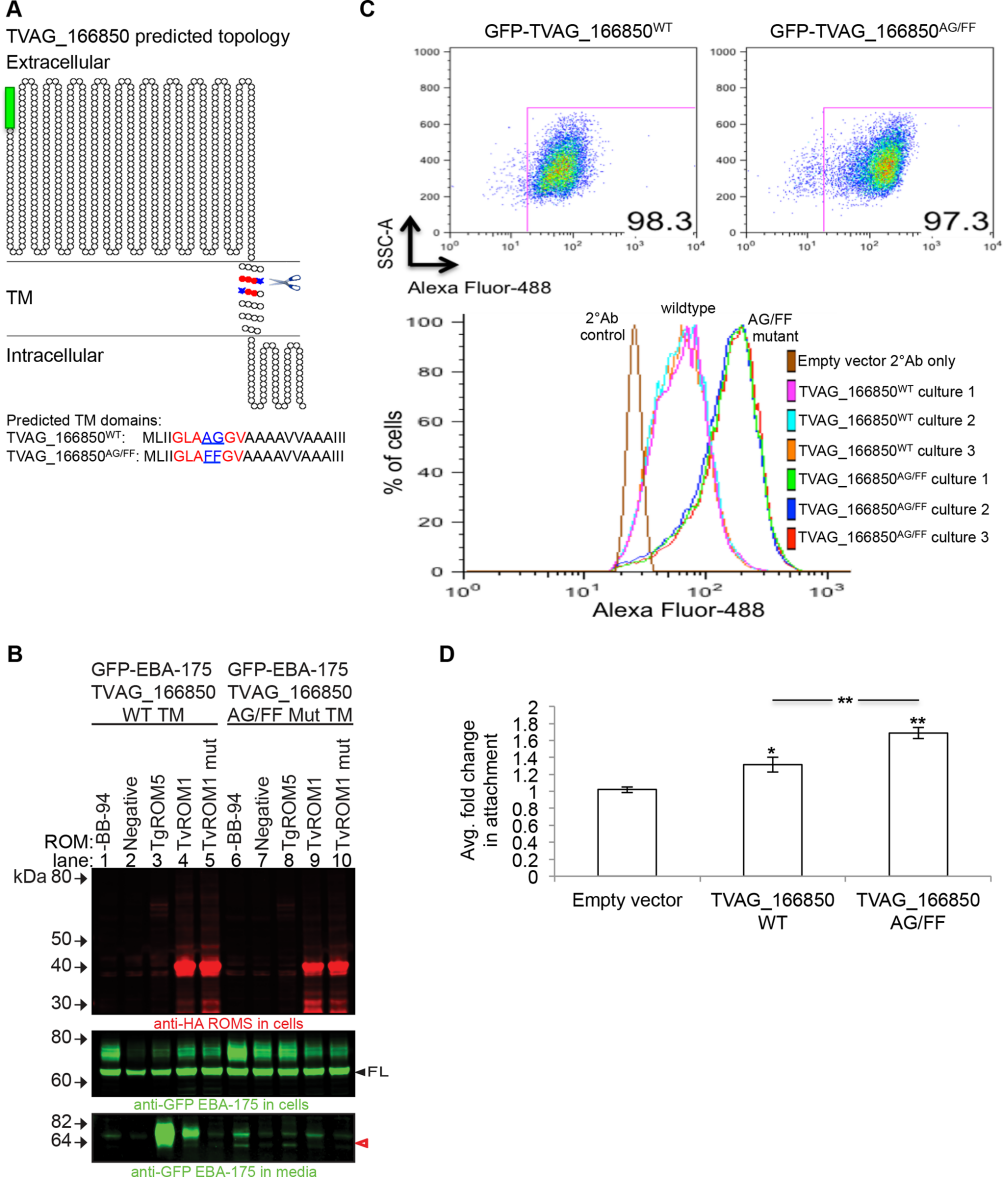
Phenotypic analysis of predicted rhomboid cleavage site mutation in the putative substrate TVAG_166850. **(A)** Predicted topology of the putative substrate TVAG_166850 using the Spoctopus TM prediction program, illustrated using the TOPO2 graphical representation program. TVAG_166850^WT^ was tagged at the N-terminus with a GFP tag (green box). The predicted rhomboid cleavage site (scissors) and the predicted P1-P1’ cleavage site residues are highlighted in blue. The surrounding parasite search motif residues are highlighted in red. The predicted TM residues are shown below. A rhomboid cleavage site mutant was generated by mutating the predicted Ala-Gly P1-P1’ residues (underlined in sequence) to Phe-Phe residues to generate the GFP-TVAG_166850^AG/FF^ mutant. **(B)** TvROM1 efficiently cleaves the wild type (wt) TVAG_166850 TM and not the TVAG_166850^AG/FF^ mutant TM. Proteases and chimeras of GFP-EBA-175 with the TVAG_166850 wt TM or mut TM were co-transfected in the heterologous cell cleavage assay. Lanes 1–5 = co-transfection with wild type GFP-EBA-175-TVAG_166850^WT^ TM; lanes 6–10 = co-transfection with GFP-EBA-175-TVAG_166850^AG/FF^ mutant TM. Western blot analyses of whole cell lysates confirm expression of wt and mut rhomboid proteases (top panel) and the full length substrates (FL, middle panel). Analyses of conditioned media show a GFP-tagged cleavage product in the media (red, open arrowhead, bottom panel) of TvROM1 co-transfected with wild type TVAG_166850 (lane 4) which is reduced ~90% in mutant TVAG_166850^AG/FF^ co-transfectants (lane 9). Co-transfection with mutant TvROM1 abolishes cleavage (lanes 5 & 10). Lanes 1 & 6 = no metalloprotease inhibitor BB-94, lane 2–5 & 7–10 = 10 μM metalloprotease inhibitor BB-94. Lanes 3 & 8 = co-transfection with TgROM5 (control). **(C)** The GFP-TVAG_166850^WT^ and GFP-TVAG_166850^AG/FF^ mutant proteins were exogenously expressed in *T*. *vaginalis*. Transfectants were stained with an anti-GFP antibody without permeabilization at 4°C to detect surface levels of the fusion protein quantified using flow cytometry. Three cultures (cultures #1–3) in three independent experiments were analyzed. Representative results from one experiment are shown. Top panel shows the GFP^+^ cell population, similar percentages of GFP^+^ cells were detected for the wt and mutant transfectants. Bottom histogram shows the fluorescence intensity distribution of the GFP^+^ population. GFP-TVAG_166850^AG/FF^ mutant transfectants had at least three-fold higher mean fluorescence intensity (MFI) levels compared to wild type transfectants. **(D)** The ability of GFP-TVAG_166850^WT^ and GFP-TVAG_166850^AG/FF^ mutant transfectants to attach to ectocervical cells was compared to empty vector transfectants. Results show the average of three experiments, each conducted in triplicate. Exogenous expression of GFP-TVAG_166850^WT^ leads to a statistically significant increase in attachment compared to empty vector transfectants (*p<0.05). Overexpression of the GFP-TVAG_166850^AG/FF^ mutant leads to an even greater increase in attachment compared to empty vector (**p<0.01) and wildtype GFP-TVAG_166850^WT^ transfectants (**p<0.01).

We have previously reported that the TvROM1 substrate identified in this study, TVAG_166850, increases parasite attachment to host cells when exogenously expressed in a poorly adherent *T*. *vaginalis* strain [23]. To address whether cleavage by TvROM1 influences TVAG_166850’s ability to mediate host cell attachment we introduced mutations at the predicted rhomboid cleavage site. The predicted P1-P1’ site Ala672-Gly673 (AG) residues were mutated to Phe-Phe (FF) residues, since we previously found that mutation of small amino acids at the predicted cleavage site to bulky Phe significantly decrease rhomboid cleavage of the *T*. *gondii* AMA1 substrate [38]. Fig 6A shows a graphical representation of GFP-TVAG_166850 and the predicted rhomboid cleavage site. To verify that the mutations introduced into the GFP-TVAG_166850^AG/FF^ mutant affect TvROM1 cleavage, the HEK293 heterologous cell cleavage assay was used to compare TvROM1 cleavage of the chimeric EBA-175 protein containing either the wild type or mutant TM domain of TVAG_166850. The GFP-EBA-175-TVAG_166850TM^AG/FF^ mutant chimeric protein was cleaved 90% less than the chimeric protein containing the wild type TM domain (Fig 6B-lane 4 *vs*. lane 9). Cleavage of the wild type and mutant TM domains, above background levels, was not observed using the His316Ala mutant of TvROM1 (Fig 6B-lane 5 and lane 10). Similarly, the *Toxoplasma gondii* TgROM5 that can also cleave the wild type TM domain of TVAG_166850 (Fig 6B-lane 3) and exhibited limited cleavage of the mutant TM domain (Fig 6B-lane 8).

To test whether surface substrates of TvROM1 play a role in host: pathogen interactions, N-terminally tagged GFP-TVAG_166850^WT^ or the GFP-TVAG_166850^AG/FF^ mutant were exogenously expressed in the poorly adherent *T*. *vaginalis* strain G3. The levels of wild type and mutant proteins expressed and presented on the surface of transfectants were then measured using flow cytometry. The percent of cells expressing the GFP-tagged proteins were similar in the GFP-TVAG_166850^WT^ and GFP-TVAG_166850^AG/FF^ mutant populations (Fig 6C, top panel). However, the mean fluorescence intensity (MFI) of the GFP-TVAG_166850^AG/FF^ mutant population was consistently at least three-fold higher than the wild type population in three independent experiments (a representative experiment is shown in Fig 6C, bottom panel). The increased MFI signal observed in the GFP-TVAG_166850^AG/FF^ population continued to be observed after growing the parasites in culture for several days and after freeze-thawing of the population, providing evidence for a robust and stable phenotype. Higher surface staining of the rhomboid substrate MIC2 in *T*. *gondii* rhomboid knockout cells has also been reported by Shen *et al*. [62]. Our data indicate that increased surface detection of the GFP-TVAG_166850^AG/FF^ mutant protein results from lack of cleavage by TvROM1, allowing for its greater accumulation at the cell surface.

To determine the phenotypic effect of mutating the predicted rhomboid cleavage site in GFP-TVAG_166850, we compared the attachment properties of the GFP-TVAG_166850^WT^ and GFP-TVAG_166850^AG/FF^ mutant populations. Exogenous expression of GFP-TVAG_166850^WT^ led to a 1.3-fold increased attachment to host cells compared to empty vector transfectants (p-value<0.05), while exogenous expression of the GFP-TVAG_166850^AG/FF^ mutant led to an even greater increase in attachment (1.7-fold) compared to empty vector (p-value<0.01) (Fig 6D). The change in attachment between the mutant and wild type was statistically significant (p-value<0.01). These data indicate that reduced TvROM1 cleavage leads to the increased surface levels of the GFP-TVAG_166850^AG/FF^ mutant protein (Fig 6C) which in turn leads to increased attachment to host cells (Fig 6D). Incubation of GFP-TVAG_166850^WT^ and GFP-TVAG_166850^AG/FF^ mutant populations with ectocervical cells in a four hour cytolysis assay did not reveal a greater ability of the transfectants to lyse host cells (S5 Fig), indicating additional factors likely modulate *T*. *vaginalis* cytolysis of host cells.

## Discussion

We have characterized three putative rhomboid proteases in the human parasite *T*. *vaginalis* and demonstrated that two, TvROM1 and TvROM3, are catalytically active and exhibit distinct substrate specificity. TvROM3 cleaves the canonical rhomboid substrate Spitz in a heterologous cell cleavage assay while TvROM1 does not cleave Spitz but does cleave several known *Plasmodium* rhomboid substrates [39, 44]. Immunofluorescence studies of exogenously expressed HA-tagged TvROM1 and TvROM3 indicate that TvROM1 is a plasma membrane protease whereas TvROM3 localizes to Golgi-like structures. Cellular localization studies of the endogenous proteins are yet to be done to corroborate the localization determined for the HA-tagged proteins.

As a step towards defining the function of TvROMs, we identified nine putative *T*. *vaginalis* surface substrates using both quantitative proteomic and bioinformatic approaches. Two of these candidate substrates were shown to be TvROM1 substrates: TVAG_166850 and TVAG_280090. These proteins have a high degree of similarity and are members of a gene family consisting of over 150 members [20]. Interestingly, two other predicted substrates (TVAG_244130 and TVAG_335250) are paralogs of TVAG_166850 and TVAG_280090; however their TM domains were not cleaved by TvROM1 in the heterologous cell cleavage assay. Whether these putative substrates are cleaved by other proteases not examined in this study remains to be determined. TvROM3 was not able to cleave any of the nine putative *T*. *vaginalis* substrates identified, confirming that TvROM1 and TvROM3, which display different subcellular localizations, also appear to have different substrate specificities.

We were not able to identify *T*. *vaginalis* substrates for either TvROM2 or TvROM3. However, given that the candidate substrates identified are members of large protein families, untested members of these families or other unidentified proteins may serve as substrates for these TvROMs. This is consistent with our observation that *T*. *vaginalis* surface proteomes contain a subset of proteins encoded by large multi-gene families, with different subsets expressed by different strains [23].

Amino acids surrounding the predicted cleavage sites in model rhomboid substrates cleaved by TvROM1 and the *T*. *vaginalis* substrates are similar. There is an overall presence of small amino acid residues surrounding the predicted cleavage site and indeed restriction of a small amino acid at the P4 and P2’ sites in our parasite search motif was critical for bioinformatic identification of the two *T*. *vaginalis* substrates cleaved by TvROM1. Nevertheless, finding that TvROM1 cleaves only two of the five candidate substrates identified by bioinformatics underscores the difficulty in identifying substrates using search motifs. Overall, these analyses indicate that TM dynamics may play a more important role in substrate selection than the presence of specific amino acids surrounding the cleavage site.

We found that overexpression of TvROM1 significantly increases parasite attachment to and lysis of host cells, which are two properties critical for the pathogenesis of *T*. *vaginalis*. These data may be explained several ways. Signaling induced by cleavage of a TvROM substrate during the initial phase of parasite-host interaction may lead to an increase in adherent surface proteins on the parasite or host cell. This might be mediated by an initial low-affinity binding, with cleavage driving a higher affinity binding, which in turn triggers host cell lysis. A role for TvROM1 at the junction of adherence and cytolysis is consistent with the observation that once a threshold of parasite attachment is reached, host cell cytolysis is triggered [17]. Exogenous expression of the TVAG_166850 substrate with the predicted P1-P1’ rhomboid cleavage site mutated to reduce TvROM1 cleavage leads to an even greater increase in parasite attachment relative to exogenous expression of the wild type protein, consistent with a direct role for this substrate in adherence and regulation of its function by TvROM1 cleavage. In this regard, it is notable that using the Phyre2 program, both TVAG_166850 and TVAG_280090 have cadherin-like predicted secondary structures which are known to mediate cell: cell interactions in eukaryotes [63].

The proteins identified in supernatants of *T*. *vaginalis* (S1 Table) together with proteins we previously identified to be released in exosomes [64], compose, to our knowledge, the most comprehensive analyses lists of proteins released/secreted by *T*. *vaginalis* to date. There is overlap between these two proteomes, with 70 of the 188 proteins identified here also being present in the *T*. *vaginalis* exosome proteome (S1 Table). We identified cysteine proteases, which are considered to be established and secreted virulence factors of *T*. *vaginalis* (reviewed in [65]). This list also provides experimental evidence for the release of proteins previously predicted to be *T*. *vaginalis* secreted proteins, such as cysteine protease inhibitors called cystatins [66] and a pore-forming/saposin-like protein hypothesized to help lyse host cells [20, 21, 67]. Other soluble factors uncovered in this proteomic analysis may be candidates contributing to *T*. *vaginalis* virulence.

In summary, we have identified two active *T*. *vaginalis* rhomboid proteases (TvROM1 and TvROM3) and two TvROM1 substrates. We have shown that modulation of expression of TvROM1 and its substrate plays a role in regulating host cell attachment and cytolysis dynamics. These findings are significant as few proteins have been shown to contribute to both *T*. *vaginalis* attachment and cytolysis of host cells (reviewed in [16]). The results of this study also expand the finding that rhomboid proteases contribute to pathogenesis-associated processes in parasites that pose important public health problems (reviewed in [68] and [69]). As both human and TvROM1 substrate specificities are better defined, it may be possible to selectively target *T*. *vaginalis* rhomboid proteases as therapeutic agents [70].

## Materials and Methods

### Growth of cells

The *T*. *vaginalis* strains G3 (ATCC PRA98) and RU393 (ATCC 50142) were grown as described previously [71]. Parasites were incubated at 37°C and passaged daily for less than two weeks. The human ectocervical cell line Ect1 E6/E7 (ATCC CRL-2614) was grown as described [72].

### TvROMs plasmid construction and exogenous expression in *T*. *vaginalis*

TvROMs were PCR amplified from G3 genomic DNA, using the primer pairs listed in S6 Fig. PCR fragments were cloned into the Nt-HA-MasterNeo plasmid [23]. To increase the detection of tagged TvROMs, two additional HA tags were inserted to generate 3xHA-TvROMs by ligation of hybridized oligos encoding two HA-tags into the NdeI restrictions sites. The 3xHA-TvROM-MasterNeo plasmid was then digested with ClaI to remove the fragment encoding the neomycin phosphotransferase (Neo) selectable marker and its flanking 5′ and 3′ beta-tubulin untranslated regions (UTRs) and ligated to close the vector. A fragment encoding the puromycin N-acetyltransferase gene flanked with alpha-SCS 5′ and 3′ UTRs previously described in [73] was cloned into the Master (-Neo) plasmid using the ApaI restriction site.

Electroporation of the G3 and RU393 strains was performed as previously described [74] and transfectants were selected with 100 μg/ml G418 (GIBCO) or 60 μg/ml puromycin dihydrochloride (A.G. Scientific, Inc.) and maintained with drug selection.

### TvROMs indirect immunofluorescence assays

The subcellular localization of 3xHA-TvROM1 in RU393 transfectants was determined by cell surface biotinylation of transfectants followed by IFA and imaging as described [23] except for use of fewer parasites, 3.18×10^7^ parasites in 30 ml. For localization of 3xHA-TvROM2 and 3xHA-TvROM3 in G3 transfectants, IFA was performed as described [75].

### HEK293 heterologous cell cleavage assays and PfEBA-175 cleavage site determination

The *in vitro* activities of TvROMs were analyzed with a heterologous cleavage assay derived from [39]. Briefly, HEK293T (ATCC CRL-11268) cells were seeded in 12-well plates and co-transfected at ~80% confluency with X-tremeGENE HP (Roche) and pcDNA3.1 (Invitrogen) expressing N-terminal GFP-tagged and/or C-terminal Flag-tagged substrates and N-terminal 3xHA-tagged rhomboid proteases. Cells were washed once and then conditioned with serum-free DMEM 18-h post-transfection. Media and cell lysates were harvested 24-h later in Laemmli buffer. Protein from lysates and media were separated on 4%-12% gradient Bis-Tris and 4%-20% Tris-glycine gels, respectively, (Life Technology) and transferred onto nitrocellulose membranes. Membranes were probed with anti-GFP (Abcam), anti-HA (Roche), or anti-FLAG (Sigma) primary antibodies, anti-rabbit/rat/mouse secondary antibodies conjugated to infrared fluorophores (Li-COR Biosciences) and imaged on an Odyssey infrared scanner (Li-COR Biosciences). For cleavage site determination, HEK293T cells were transfected as described, lysed in RIPA buffer, subjected to anti-FLAG immunopurification (Sigma) and analyzed by MALDI-TOF mass spectrometry as previously described [76]. TvROMs were amplified from G3 genomic DNA via PCR and cloned into the 3xHA pcDNA expression vector. Like other parasitic rhomboid enzymes [39], TvROMs did not express in HEK293T cells and were thus recoded for human expression (GeneArt).

### TvROMs cytolysis and attachment assays

*T*. *vaginalis* adherence and cytolysis assays were performed as described [17]. To test the effect of 3,4-dichloroisocoumarin (3,4-DCI, SIGMA) on parasite adherence, parasites were incubated with 3,4-DCI or DMSO vehicle control in completed K-SFM media and added to ectocervical cell monolayers. Adherence and cytolysis assays were performed as described [17], using three or four hour incubation times for wildtype RU393 and RU393 3x-HA-TvROM1 transfectants, respectively.

### Stable isotope dimethyl labeling of cell supernatants and quantitative proteomics

1×10^7^ log-phase 3xHA-TvROM1 RU393 *T*. *vaginalis* transfectants in 100 ml of PBS+5% sucrose were treated with 50 μM 3,4-DCI or DMSO vehicle control for one hour at 37°C. Parasites were pelleted and the supernatant was filtered through a 0.22 μm Steriflip**™** 50 ml filter (Millipore) and stored at -80°C. Upon thawing, supernatants were concentrated to ~300 μl using an Amicon® Ultra Centrifugal filter unit (3 kDa cutoff) and lyophilized. 25 μg of protein from each sample was reduced, alkylated, and digested with Lys-C endopeptidase (Wako, Richmond, VA) for four hours and trypsin for 14 hours (Thermo Scientific) as previously described [77]. Digested peptides were desalted by HPLC using a Michrom Bioresources Microtrap column (2–20 μg binding capacity). Stable isotope dimethyl labeling was performed as described [59], with the addition of an HPLC desalting step after peptide labeling followed by lyophilization. Peptides from the vehicle control were labeled with the “light label” and 3,4-DCI samples were labeled with the “intermediate label.” Labeled peptides were reconstituted in 0.2% formic acid, and equal protein amounts were mixed. For mass spec analysis, 8 μl of a mixture containing 1 μg of each sample was separated on an analytical (75 μm ID) HPLC column packed in-house with ReproSil-Pur C_18_AQ 3 μm resin (120 Å pore size, Ammerbuch, Germany) coupled to an LTQ-Orbitrap Classic mass spectrometer (Thermo Scientific, Bremen, Germany) as described [78] with the following modifications. The nanoflow LC system EASY nLC II gradient was as follows: 2–30% B (80 min), 30–100% B (1 min) and 100% B (8 min).

### MS data analysis

MaxQuant (v. 1.4.1.2) was used to search Thermo RAW files [79]. Spectra were searched against *T*. *vaginalis* (50623 entries) and a contaminant database (245 entries). MaxQuant generated decoy sequences (reversed peptide sequences) to estimate the false discovery rate. Search parameters included variable oxidation of methionine, variable protein N-terminal acetylation, fixed carboxyamidomethylation of cysteine and dimethyl labeling of peptide N-terminus and lysine. Trypsin was specified as the digestion enzyme with up to two missed cleavages. A 1% false discovery rate threshold was applied for protein and peptide identifications. Precursor mass tolerance was 7 ppm (or less for individual peptides). Fragment mass tolerance was 0.5 Da. Confidence intervals and p-values were calculated using a hierarchical model with bootstrap resampling and pooled variance estimates as described [80]. P-values were adjusted using the Benjamini and Hochberg method to correct for multiple hypothesis testing [81].

### Heterologous cell cleavage assays

The transmembrane domains from candidate substrates were inserted into EBA-175 by inverse PCR (Phusion, NEB) with primers encoding the new transmembrane domains at their 5′ ends. PCR products were purified (QiaQuick, Qiagen) and blunt ends were phosphorylated by T4 polynucleotide kinase (NEB), and ligated with T4 ligase (Roche). The chimeric molecules were confirmed by DNA sequencing and analyzed for cleavage in the heterologous cleavage assay as described above.

### Site-directed mutagenesis, expression, and phenotypic analysis of GFP-TVAG_166850^WT^ and rhomboid cleavage-site mutant GFP-TVAG_166850^AG/FF^

The plasmid encoding for TVAG_166850 with an N-terminal enhanced green fluorescent protein (eGFP) tag was constructed by cloning a eGFP PCR-amplified fragment into the NdeI and SacII restriction sites of Nt-HA-MasterNeo, followed by ligation of the TVAG_166850 gene in-frame using the SacII and BamHI restriction sites. The TVAG_166850^AG/FF^ putative rhomboid cleavage site mutant was generated by site-directed mutagenesis of the GFP-TVAG_166850 construct using the QuikChange kit (Stratagene) and primers that encoded the Ala672Phe and Gly673Phe mutations (primers listed in S6 Fig). Plasmids were transfected into the poorly adherent G3 strain for gain of function analysis, and selected with G418. Attachment to and cytolysis of host cells was assayed as described above except the cytolysis assay was incubated for 18 hrs.

### Flow cytometry analysis of surface GFP-TVAG_166850^WT^ and GFP-TVAG_166850^AG/FF^ mutant proteins

2×10^5^ log-phase G3 transfectants were resuspended in FACS buffer (5% fetal bovine serum/0.1% sodium azide/1× PBS) and assayed in triplicate. Parasites were stained with 1 μg/ml mouse-anti-GFP (Clontech) and Empty vector transfectants were stained with only the secondary (2°) Ab as a control for background. Staining was detected with 1 μg/ml goat anti-mouse-Alexa Fluor^**®**^488 2°Ab antibody. Antibody incubations were performed on ice for 30 min. Samples were analyzed on a Becton Dickinson Fortessa flow cytometer in the FITC channel. Empty vector 2°Ab alone samples were used to set the GFP^+^ population gates. The mean fluorescence values were calculated for each sample, using the FlowJo 8.4 software. The experiment was performed three times and representative data from one experiment is shown.

## Supporting Information

S1 Fig Alignment of TvROM 1–4 and predicted membrane topology. **(A)** Sequence alignment of the predicted active TvROMs 1–4 using the Multiple Sequence Comparison by Log-Expectation (MUSCLE) program. Alignment was manually edited using the BioEdit program (“~” marks sites of manual insertion/deletion to aid alignment of critical residues, numbers within the sequences indicate the number of residues omitted to allow optimal alignment). Shading indicates 75% or greater sequence similarity. Lines above the TvROM1 sequence marks predicted TM domains for TvROM1 using the Spoctopus signal peptide and membrane protein topology prediction program. Similar TM domain predictions were obtained for TvROM2 and TvROM4. TvROM3 lacks a predicted 7^th^ TM domain and thus the C-terminal tail is predicted to be intracellular. No signal peptides were predicted for TvROMs 1–4, and all the proteins are predicted to have their N-terminus located inside the cell. Bold red letters indicate residues participating in nucleophilic catalysis, while the green residues function in electrophilic catalysis (oxyanion stabilization). Blue lettering highlights residues that form four conserved Keystones that play key roles in maintaining rhomboid architecture (as defined experimentally for bacterial rhomboid proteases in R.P. Baker & S. Urban, Nature Chemical Biology, 2012). Briefly, the arginine (R) of Keystone I (E/QxWRxxS/TxxxxH) helps to stabilize the L1 loop ‘hairpin’ by donating several hydrogen bonds to neighboring residues, while GxxxExxxG of Keystone II stabilizes cytoplasmic interaction of TMs 1, 2 and 3 into an apex. Keystone III (surrounding the catalytic serine) and Keystone IV (with a GxxxG dimerization motif following the catalytic histidine base) mediate close apposition of TMs 4 and 6 at the core of the enzyme. **(B)** Scheme shows the predicted topology of TvROM1, TvROM2, and TvROM4 with 7 TM domains. TvROM3 has similar predicted topology but contains only the first six TM domains. Reference: R.P. Baker and S. Urban. Architectural and thermodynamic principles underlying intramembrane protease function. Nat Chem Biol. 2012: 8(9):759–768.
(TIFF)

S2 Fig Additional cleavage activity analysis of TvROM1-3 using heterologous substrates. The ability of *T*. *vaginalis* rhomboid proteases to cleave known model rhomboid substrates was tested using the HEK293 heterologous cell cleavage assay. Proteases were HA tagged and substrates contained an N-terminal GFP tag to allow detection. Whole cell lysates (WCL) and conditioned media (CM) were collected from co-transfectants and analyzed by Western blot analyses (Baker *et al*. 2006). **Top panels**: rhomboid protease detected in WCL using an anti-HA antibody; **middle panels**: full-length (FL-filled arrowheads) and cleaved substrates (open arrowheads) detected in WCL using an anti-GFP antibody; **bottom panels**: cleaved substrate fragments detected in CM using an anti-GFP antibody (red, open arrowheads). The substrates tested were **(A)** *Plasmodium falciparum* BAEBL, **(B)** human Ephrin-B3, **(C)** *Plasmodium falciparum* EBA-175, **(D)** *Drosophila melanogaster* Spitz, and **(E)** *Providencia stuartii* TatA. The positive control protease for testing cleavage of Spitz and TatA **(D** and **E)** was DmRho1; the positive control protease for *Plasmodium* BAEBL **(A)** and EBA-175 **(C)** was PfROM4; the positive control protease for EphrinB3 was TvROM1 **(B)**. Negative controls lacked co-transfection with a TvROM (Negative). TvROM1/TvROM2/TvROM3 = wild type protease; TvROM1 mut/TvROM2 mut/TvROM3 mut = protease with the catalytic histidine mutated to alanine. In **(B)** the heterologous cell cleavage assay was performed in the absence and presence of 10 μM Batimastat (- or + BB-94), a metalloprotease inhibitor. TvROM1 can cleave *Plasmodium* BAEBL (A-lane 3) and the TvROM1mut cannot (A-lane 4) indicative of TvROM-1 specific cleavage. EphrinB3 and TatA are released into the media by both TvROM2 and TvROM2mut co-transfectants (B-lanes 3 and 4 and E-lanes 4 and 5), therefore it does not appear to be TvROM2-specific cleavage. To further investigate this, TvROM2 cleavage of EphrinB3 was tested in the presence of a metalloprotease inhibitor and release of EphrinB3 above background was no longer observed (B-lanes 7 and 8). **References:** Baker, R. P., R. Wijetilaka and S. Urban (2006). PLoS Pathog 2(10): e113.
(TIF)

S3 Fig TvROM1 and TvROM3 are not able to cleave remaining putative substrates from dimethyl labeling mass spectrometry experiments. The HEK293 heterologous cell cleavage assay was used to test cleavage of the TM domain of the putative substrates identified in the quantitative proteomics experiment (Fig 4). A plasmid encoding a chimeric protein composed of GFP-*P*. *falciparum* EBA-175 with the TM domain replaced with that of the putative substrates, was co-transfected with a plasmid encoding for wild type HA-TvROM1 or HA-TvROM3, or catalytic His to Ala mutants (mut). The chimeric protein tested is indicated above each blot. Negative controls lacked co-transfection with a TvROM (Negative). Western blot analysis of whole cell lysates (WCL) and conditioned media (CM) from co-transfectants was performed with an anti-GFP antibody to test for the presence of a smaller GFP-EBA-175 fragment released into the media by TvROM1 cleavage (bottom panel) or detected in cell lysates if cleaved by TvROM3 (middle panel). An open red arrowhead marks the location of the expected molecular weight for the cleavage product if cleavage had occurred. An anti-HA antibody was used to confirm expression of TvROM1 and TvROM3 wt and mut proteins (top panel). Full-length (FL) chimeric substrate in WCL is annotated with a filled arrowhead (middle panel). The chimeric protein EBA-175 with the TM domain of TVAG_573910 **(A)** was released into the media even in the absence of co-transfection with TvROM1 or TvROM3 (A-lane 1, bottom panel), therefore the heterologous cell cleavage assay was performed in the presence of 10 μM Batimastat (A-lanes 2–7), a metalloprotease inhibitor. Release of the chimeric protein was still detected when no TvROM was co-transfected and in the presence of the inhibitor (A bottom panel-lane 2), thus we do not consider the products released into the media (bottom panel) to be generated by TvROM-specific cleavage. No differences in the amount of cleavage product could be detected for the other substrates **(B)** TVAG_245580, **(C)** TVAG_425470, and **(D)** TVAG_393390) between wildtype and catalytic mutants and thus do not consider the products to be generated by rhomboid protease-specific cleavage.
(TIF)

S4 Fig Additional cleavage analysis of putative surface proteome substrates. The HEK293 heterologous cell cleavage assay was used to test the cleavage of the TM domain of the putative substrates identified in the screen of the surface proteome with the parasite search motif (Fig 5). A plasmid encoding a chimeric protein composed of GFP-*P*. *falciparum* EBA-175 with the TM domain replaced with that of the putative substrates **(A-E)**, was co-transfected with a plasmid encoding for wildtype HA-TvROMs 1–3 or catalytic His to Ala mutants (mut). The chimeric protein tested is indicated above each blot. Negative controls lacked co-transfection with a TvROM (Negative). Western blot analysis of whole cell lysates and conditioned media from co-transfectants was performed with an anti-GFP antibody to test for the presence of a smaller GFP-EBA-175 fragment released into the media by TvROM1 cleavage or detected in cell lysates if cleaved by TvROM2 or TvROM3. An anti-HA antibody was used to confirm expression of the HA-TvROMs wt and mut proteins (top panel). Full-length chimeric substrate is annotated with a filled arrowhead (FL-middle panel). A red open arrowhead marks the expected molecular weight of the cleavage product. As positive controls, the cleavage of TVAG_166850 and TVAG_280090 by TvROM1 is shown (A and B-lanes 2 and 3). The chimeric protein EBA-175 with the TM domain of TVAG_280090 (B-lanes 4 and 5), TVAG_244130 (C-lanes 4 and 5), TVAG_335250 (D-lanes 4 and 5), and TVAG_189150 (E-lanes 4 and 5) was released into the media of HA-TVROM2 wt and catalytic mutant co-transfectants, thus these are not considered to be specific TvROM2-generated cleavage products. **(F)** The full length GFP-TVAG_280090 protein can be cleaved by DmRho1 and detected in whole cell lysates (middle panel-lane 4, open arrowhead) and conditioned media (bottom panel, lane 4, red arrowhead). White line denotes cutting out of extraneous lanes in the blot.
(TIF)

S5 Fig Predicted rhomboid cleavage site mutation in the putative substrate TVAG_166850 does not lead to changes in host cell cytolysis. Negative control Empty vector transfectants and transfectants exogenously expressing wild type GFP-TVAG_166850 and GFP-TVAG_166850 AG/FF containing mutations in the predicted P1-P1’ cleavage site residues were incubated with ectocervical cell monolayers and host cell lysis was assessed. The average fold change in cytolysis of the transfectants expressing wild type or mutant TVAG_166850, relative to that of empty vector transfectants, is shown for four experiments performed in triplicate. Error bars denote the standard error. No statistically significant difference in host cell lysis was observed.
(TIF)

S6 Fig Primers used in this study. (PDF)

S1 Table Total list of proteins identified in the stable isotope dimethyl labeling quantitative proteomics experiments. (XLSX)

S2 Table Results from surface proteome screen using the parasite search motif. (XLS)
