# Supplementary figures and images for: A Trichomonas vaginalis Rhomboid Protease and Its Substrate Modulate Parasite Attachment and Cytolysis of Host Cells
Source: PLoS Pathog. 2015 Dec 18;11(12):e1005294. doi: 10.1371/journal.ppat.1005294 (PMC4684317; doi:10.1371/journal.ppat.1005294)

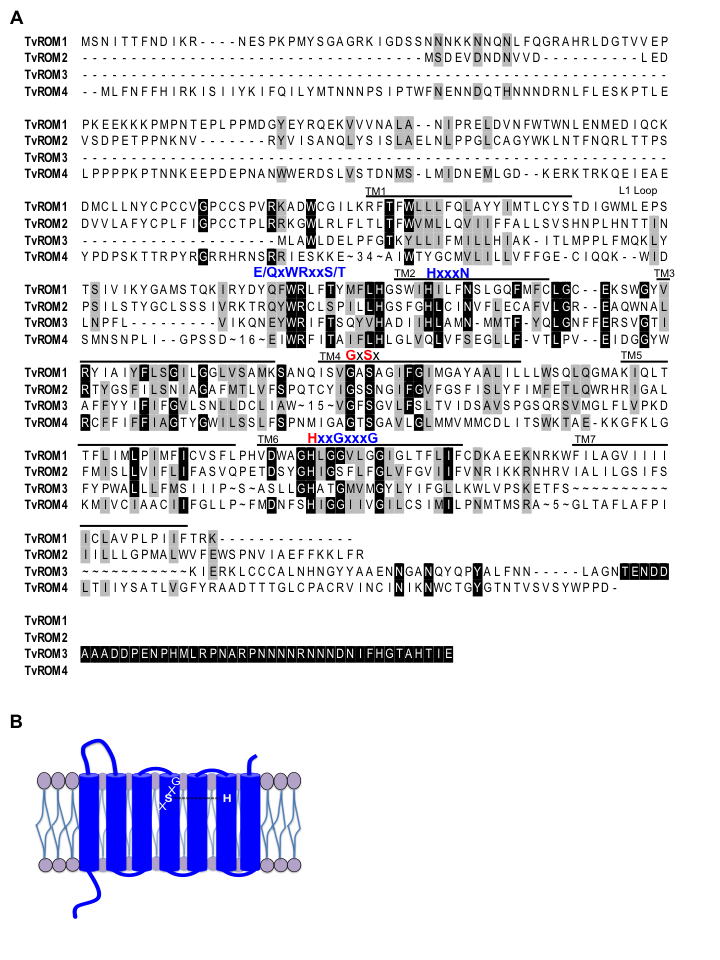

Supplement: S1 Fig — (A) Sequence alignment of the predicted active TvROMs 1–4 using the Multiple Sequence Comparison by Log-Expectation (MUSCLE) program. Alignment was manually edited using the BioEdit program (“~” marks sites of manual insertion/deletion to aid alignment of critical residues, numbers within the sequences indicate the number of residues omitted to allow optimal alignment). Shading indicates 75% or greater sequence similarity. Lines above the TvROM1 sequence marks predicted TM domains for TvROM1 using the Spoctopus signal peptide and membrane protein topology prediction program. Similar TM domain predictions were obtained for TvROM2 and TvROM4. TvROM3 lacks a predicted 7th TM domain and thus the C-terminal tail is predicted to be intracellular. No signal peptides were predicted for TvROMs 1–4, and all the proteins are predicted to have their N-terminus located inside the cell. Bold red letters indicate residues participating in nucleophilic catalysis, while the green residues function in electrophilic catalysis (oxyanion stabilization). Blue lettering highlights residues that form four conserved Keystones that play key roles in maintaining rhomboid architecture (as defined experimentally for bacterial rhomboid proteases in R.P. Baker & S. Urban, Nature Chemical Biology, 2012). Briefly, the arginine (R) of Keystone I (E/QxWRxxS/TxxxxH) helps to stabilize the L1 loop ‘hairpin’ by donating several hydrogen bonds to neighboring residues, while GxxxExxxG of Keystone II stabilizes cytoplasmic interaction of TMs 1, 2 and 3 into an apex. Keystone III (surrounding the catalytic serine) and Keystone IV (with a GxxxG dimerization motif following the catalytic histidine base) mediate close apposition of TMs 4 and 6 at the core of the enzyme. (B) Scheme shows the predicted topology of TvROM1, TvROM2, and TvROM4 with 7 TM domains. TvROM3 has similar predicted topology but contains only the first six TM domains. Reference: R.P. Baker and S. Urban. Architectural and thermodynamic princ [file ppat.1005294.s001.tiff]

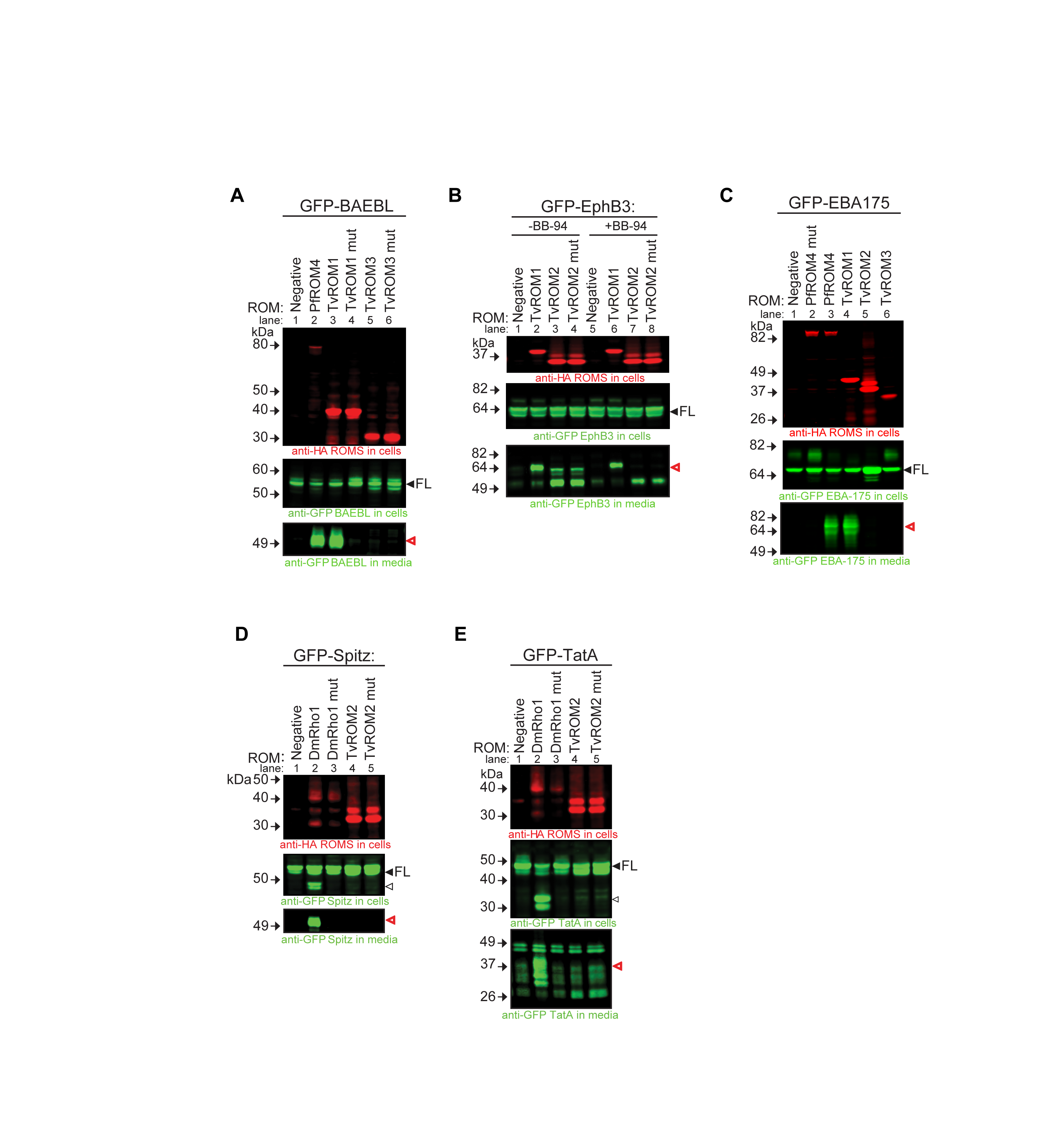

Supplement: S2 Fig — The ability of T. vaginalis rhomboid proteases to cleave known model rhomboid substrates was tested using the HEK293 heterologous cell cleavage assay. Proteases were HA tagged and substrates contained an N-terminal GFP tag to allow detection. Whole cell lysates (WCL) and conditioned media (CM) were collected from co-transfectants and analyzed by Western blot analyses (Baker et al. 2006). Top panels: rhomboid protease detected in WCL using an anti-HA antibody; middle panels: full-length (FL-filled arrowheads) and cleaved substrates (open arrowheads) detected in WCL using an anti-GFP antibody; bottom panels: cleaved substrate fragments detected in CM using an anti-GFP antibody (red, open arrowheads). The substrates tested were (A) Plasmodium falciparum BAEBL, (B) human Ephrin-B3, (C) Plasmodium falciparum EBA-175, (D) Drosophila melanogaster Spitz, and (E) Providencia stuartii TatA. The positive control protease for testing cleavage of Spitz and TatA (D and E) was DmRho1; the positive control protease for Plasmodium BAEBL (A) and EBA-175 (C) was PfROM4; the positive control protease for EphrinB3 was TvROM1 (B). Negative controls lacked co-transfection with a TvROM (Negative). TvROM1/TvROM2/TvROM3 = wild type protease; TvROM1 mut/TvROM2 mut/TvROM3 mut = protease with the catalytic histidine mutated to alanine. In (B) the heterologous cell cleavage assay was performed in the absence and presence of 10 μM Batimastat (- or + BB-94), a metalloprotease inhibitor. TvROM1 can cleave Plasmodium BAEBL (A-lane 3) and the TvROM1mut cannot (A-lane 4) indicative of TvROM-1 specific cleavage. EphrinB3 and TatA are released into the media by both TvROM2 and TvROM2mut co-transfectants (B-lanes 3 and 4 and E-lanes 4 and 5), therefore it does not appear to be TvROM2-specific cleavage. To further investigate this, TvROM2 cleavage of EphrinB3 was tested in the presence of a metalloprotease inhibitor and release of EphrinB3 above background was no longer observed (B-lanes 7 and 8). Referen [file ppat.1005294.s002.tif]

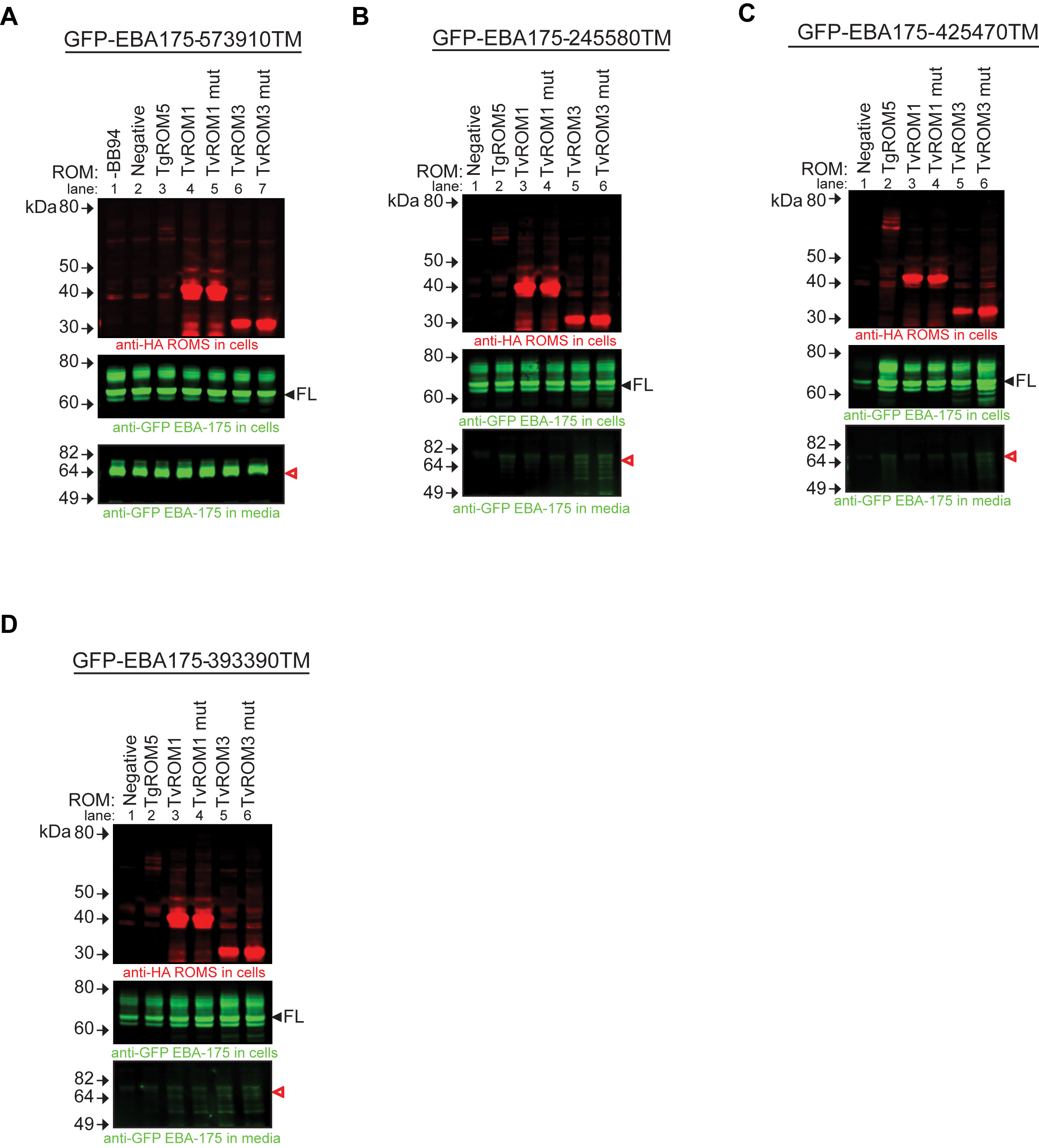

Supplement: S3 Fig — The HEK293 heterologous cell cleavage assay was used to test cleavage of the TM domain of the putative substrates identified in the quantitative proteomics experiment (Fig 4). A plasmid encoding a chimeric protein composed of GFP-P. falciparum EBA-175 with the TM domain replaced with that of the putative substrates, was co-transfected with a plasmid encoding for wild type HA-TvROM1 or HA-TvROM3, or catalytic His to Ala mutants (mut). The chimeric protein tested is indicated above each blot. Negative controls lacked co-transfection with a TvROM (Negative). Western blot analysis of whole cell lysates (WCL) and conditioned media (CM) from co-transfectants was performed with an anti-GFP antibody to test for the presence of a smaller GFP-EBA-175 fragment released into the media by TvROM1 cleavage (bottom panel) or detected in cell lysates if cleaved by TvROM3 (middle panel). An open red arrowhead marks the location of the expected molecular weight for the cleavage product if cleavage had occurred. An anti-HA antibody was used to confirm expression of TvROM1 and TvROM3 wt and mut proteins (top panel). Full-length (FL) chimeric substrate in WCL is annotated with a filled arrowhead (middle panel). The chimeric protein EBA-175 with the TM domain of TVAG_573910 (A) was released into the media even in the absence of co-transfection with TvROM1 or TvROM3 (A-lane 1, bottom panel), therefore the heterologous cell cleavage assay was performed in the presence of 10 μM Batimastat (A-lanes 2–7), a metalloprotease inhibitor. Release of the chimeric protein was still detected when no TvROM was co-transfected and in the presence of the inhibitor (A bottom panel-lane 2), thus we do not consider the products released into the media (bottom panel) to be generated by TvROM-specific cleavage. No differences in the amount of cleavage product could be detected for the other substrates (B) TVAG_245580, (C) TVAG_425470, and (D) TVAG_393390) between wildtype and catalytic mutants and thus do not [file ppat.1005294.s003.tif]

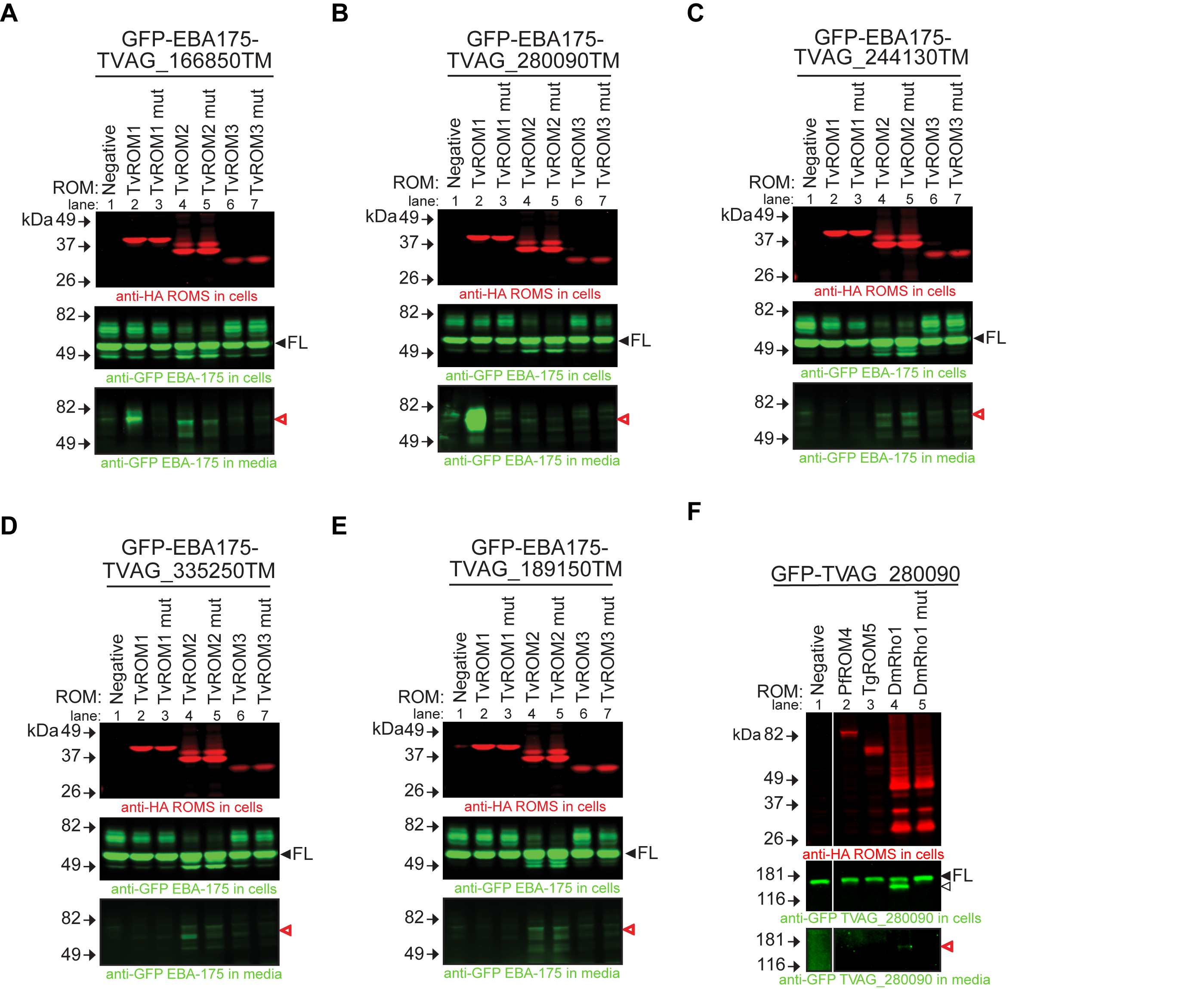

Supplement: S4 Fig — The HEK293 heterologous cell cleavage assay was used to test the cleavage of the TM domain of the putative substrates identified in the screen of the surface proteome with the parasite search motif (Fig 5). A plasmid encoding a chimeric protein composed of GFP-P. falciparum EBA-175 with the TM domain replaced with that of the putative substrates (A-E), was co-transfected with a plasmid encoding for wildtype HA-TvROMs 1–3 or catalytic His to Ala mutants (mut). The chimeric protein tested is indicated above each blot. Negative controls lacked co-transfection with a TvROM (Negative). Western blot analysis of whole cell lysates and conditioned media from co-transfectants was performed with an anti-GFP antibody to test for the presence of a smaller GFP-EBA-175 fragment released into the media by TvROM1 cleavage or detected in cell lysates if cleaved by TvROM2 or TvROM3. An anti-HA antibody was used to confirm expression of the HA-TvROMs wt and mut proteins (top panel). Full-length chimeric substrate is annotated with a filled arrowhead (FL-middle panel). A red open arrowhead marks the expected molecular weight of the cleavage product. As positive controls, the cleavage of TVAG_166850 and TVAG_280090 by TvROM1 is shown (A and B-lanes 2 and 3). The chimeric protein EBA-175 with the TM domain of TVAG_280090 (B-lanes 4 and 5), TVAG_244130 (C-lanes 4 and 5), TVAG_335250 (D-lanes 4 and 5), and TVAG_189150 (E-lanes 4 and 5) was released into the media of HA-TVROM2 wt and catalytic mutant co-transfectants, thus these are not considered to be specific TvROM2-generated cleavage products. (F) The full length GFP-TVAG_280090 protein can be cleaved by DmRho1 and detected in whole cell lysates (middle panel-lane 4, open arrowhead) and conditioned media (bottom panel, lane 4, red arrowhead). White line denotes cutting out of extraneous lanes in the blot. (TIF) [file ppat.1005294.s004.tif]

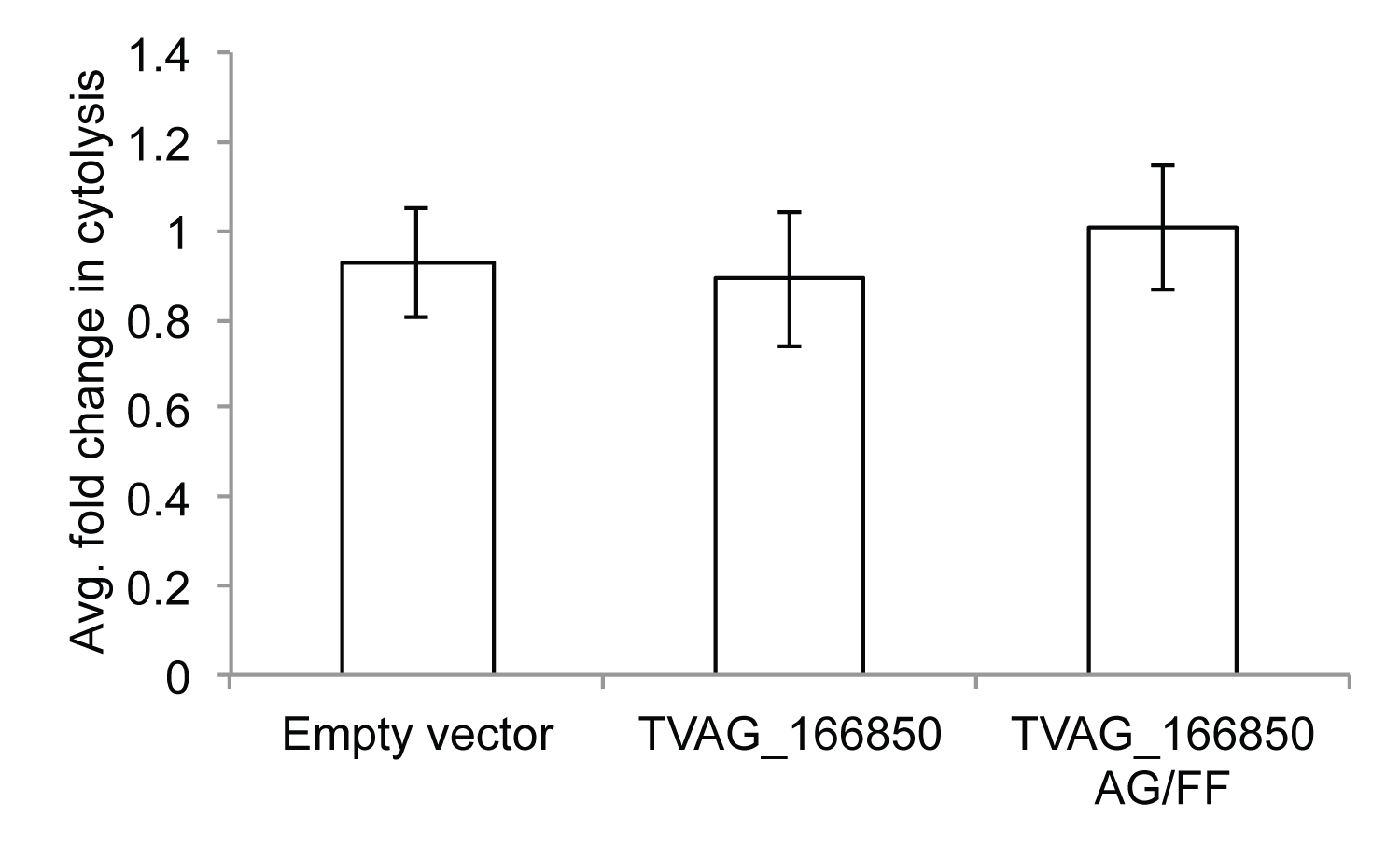

Supplement: S5 Fig — Negative control Empty vector transfectants and transfectants exogenously expressing wild type GFP-TVAG_166850 and GFP-TVAG_166850 AG/FF containing mutations in the predicted P1-P1’ cleavage site residues were incubated with ectocervical cell monolayers and host cell lysis was assessed. The average fold change in cytolysis of the transfectants expressing wild type or mutant TVAG_166850, relative to that of empty vector transfectants, is shown for four experiments performed in triplicate. Error bars denote the standard error. No statistically significant difference in host cell lysis was observed. (TIF) [file ppat.1005294.s005.tif]
