# Supplementary material for: A Trichomonas vaginalis Rhomboid Protease and Its Substrate Modulate Parasite Attachment and Cytolysis of Host Cells
Source: PLoS Pathog. 2015 Dec 18;11(12):e1005294. doi: 10.1371/journal.ppat.1005294 (PMC4684317; doi:10.1371/journal.ppat.1005294)
Supplement: S6 Fig — (PDF) [file ppat.1005294.s006.pdf]

Fig. S5: Primers used in this study.

TvROM1 amplification primers

Fwd 5’-CCGCGGATGTCGAATATTACAACCTTCAATG-3’,

Rev 5’-GGATCCTTATTTCCTTGTAAAGATAATTGGAAG-3’

TvROM2 amplification primers

Fwd 5’-CCGCGGATGAGCGACGAAGTTGATAATG-3’

Rev 5’-GGATCCTTATCTAAATAACTTCTTGAAAAATTC-3’

TvROM3 amplification primers

Fwd 5’-CCGCGGATGCTTGCGTGGCTAGATG-3’

Rev 5’-GGATCCTTATTCAATAGTATGTGCAGTACCATG-3’

Oligos for introduction of 2 HA-tags into 1x-HA MasterNeo

2HANdeFwd 5’-TATGTACCCATACGATGTTCCAGATTACGCTTACCCATACG

ATGTTCCAGATTACGCTCA-3’

2HA-NdeRev 5’-TATGAGCGTAATCTGGAACATCGTATGGGTAAGCGTAATC

TGGAACATCGTATGGGTACA-3’

TVAG_166850 amplification primers

Fwd 5’- CCGCGGATGTTACCACTATTTTACACA-3’

Rev 5’-GGATCCTTAAGCTGGGAAGATTCCTTCGAC-3’

Primers used to introduce Ala672Phe and Gly673Phe mutations in TVAG_166850

Fwd 5’- ATTATCGGCTTAGCTTTCTTCGGTGGTGTTGCCGCC-3’

Rev 5’-GGCGGCAACACCACCGAAGAAAGCTAAGCCGATAAT-3’
